# Supplementary material for: A Comprehensive Subcellular Atlas of the Toxoplasma Proteome via hyperLOPIT Provides Spatial Context for Protein Functions
Source: Cell Host Microbe. 2020 Nov 11;28(5):752–766.e9. doi: 10.1016/j.chom.2020.09.011 (PMC7670262; doi:10.1016/j.chom.2020.09.011)
Supplement: Document S1. Figures S1–S7 [file mmc1.pdf]

Cell Host & Microbe, Volume *28*

Supplemental Information

A Comprehensive Subcellular Atlas of the *Toxoplasma* Proteome via hyperLOPIT Provides Spatial Context for Protein Functions

Konstantin Barylyuk, Ludek Koreny, Huiling Ke, Simon Butterworth, Oliver M. Crook, Imen Lassadi, Vipul Gupta, Eelco Tromer, Tobias Mourier, Tim J. Stevens, Lisa M. Breckels, Arnab Pain, Kathryn S. Lilley, and Ross F. Waller

A comprehensive subcellular atlas of the *Toxoplasma* proteome via hyperLOPIT provides spatial context for protein functions

Konstantin Barylyuk, Ludek Koreny, Huiling Ke, Simon Butterworth, Oliver M. Crook, Imen Lassadi, Vipul Gupta, Eelco Tromer, Tobias Mourier, Tim J. Stevens, Lisa M. Breckels, Arnab Pain, Kathryn S. Lilley, and Ross F. Waller

Supplemental Information

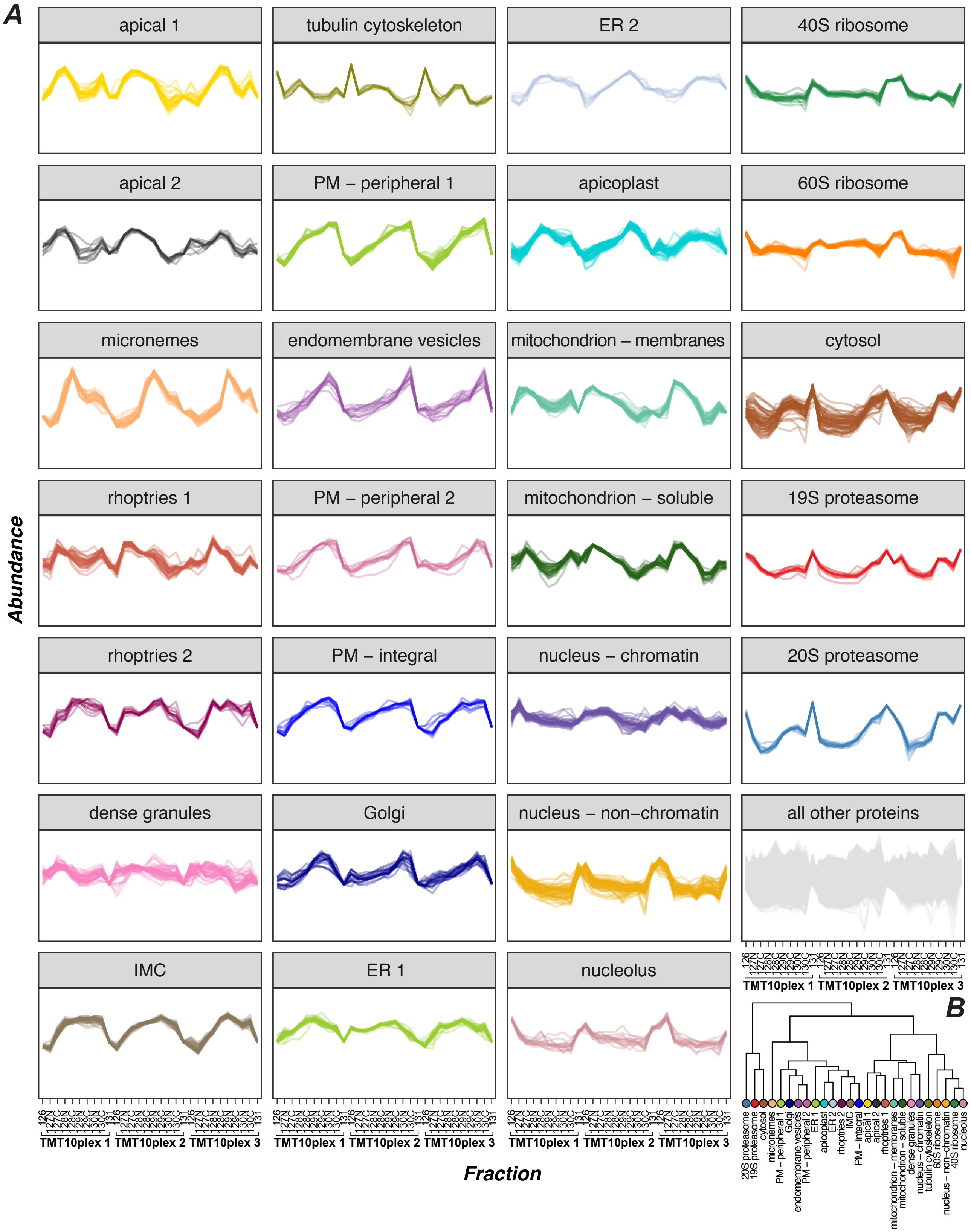

Figure S1. Abundance distribution profiles of marker and unknown proteins measured across three hyperLOPIT experiments, related to Figure 1.

A. The normalized intensities are shown on the Y-axis (Abundance). Data from three 10plex hyperLOPIT experiments are concatenated to yield a single 30plex dataset. The fractions are labelled according to the TMT10plex tag used for labelling.

B. A dendrogram of hierarchical clustering of marker protein abundance distribution profiles. For each subcellular class, a consensus abundance distribution profile was generated by averaging the profiles of the respective marker proteins. Hierarchical clustering was performed using Euclidean distance and unweighted pair group method with arithmetic mean (UPGMA)

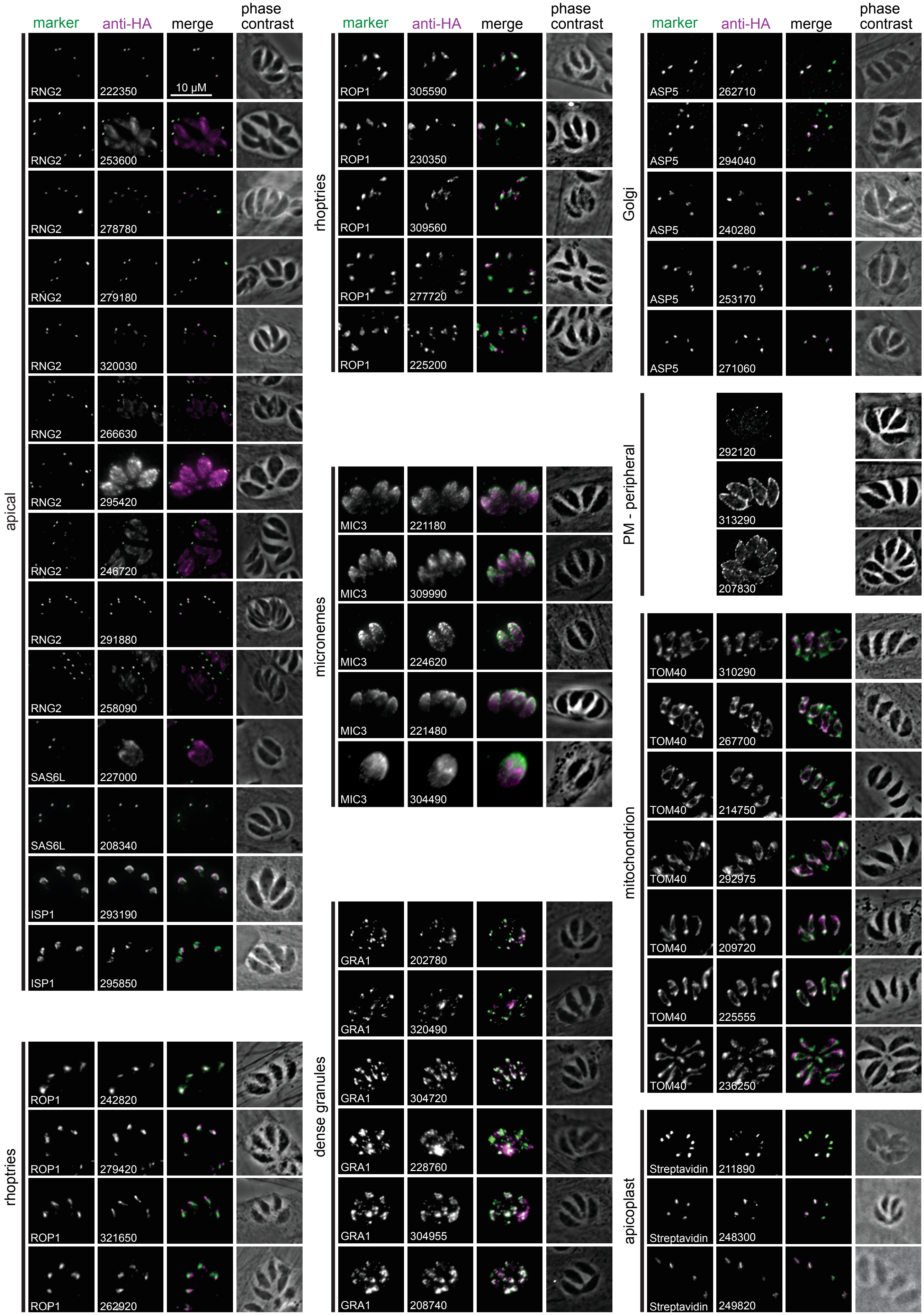

Figure S2. Validation of hyperLOPIT-predicted subcellular locations of select uncharacterized proteins by epitope tagging and immunofluorescence microscopy, continued from Figure 2.

Scale bar = 10 µm for all.

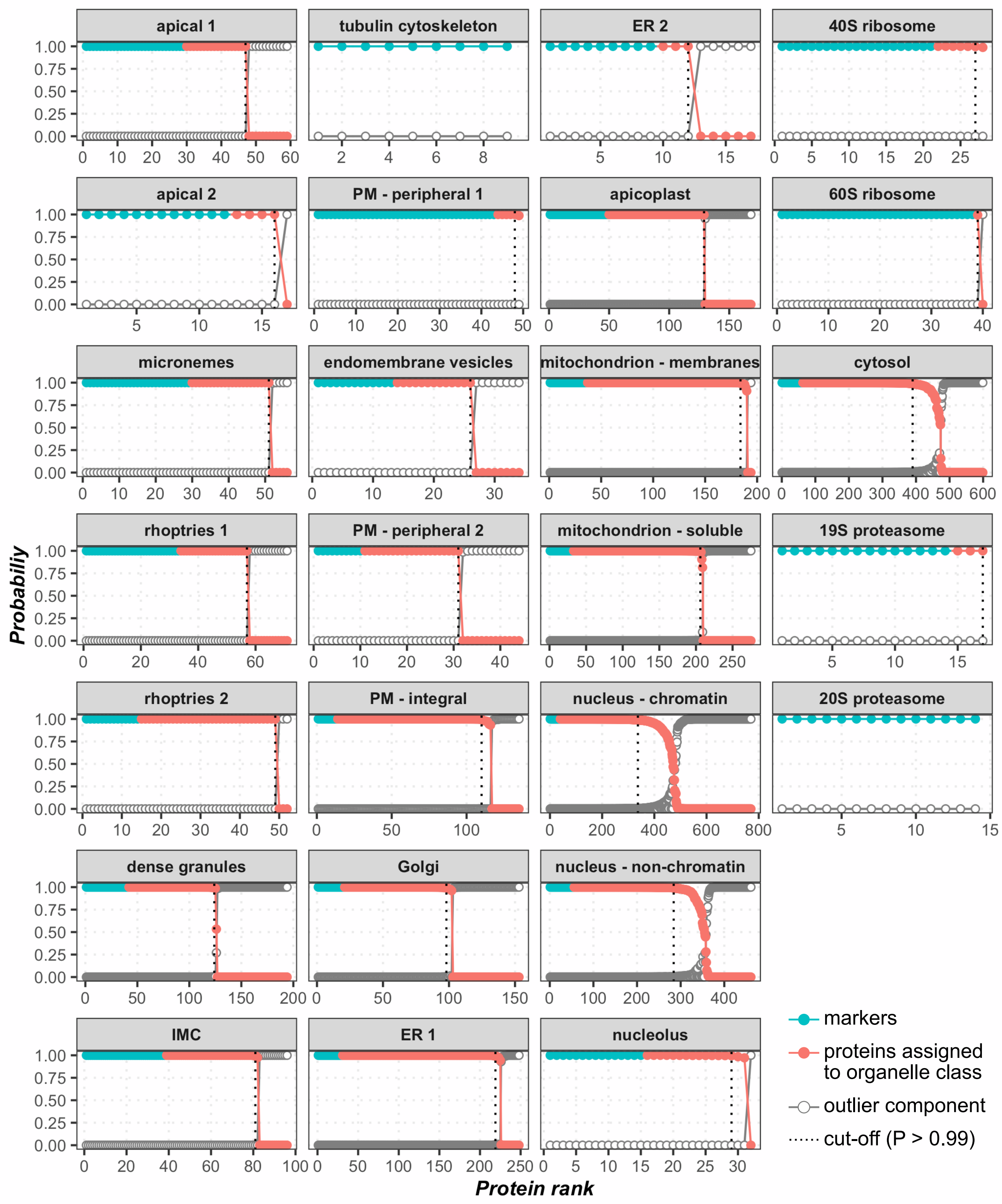

Figure S3. The posterior localization probabilities of 3,832 *T. gondii* proteins determined by a supervised Bayesian classification method TAGM-MAP, related to Figure 3.

Proteins are grouped by the most probable subcellular class, as per the TAGM-MAP classification result, and ranked on the x-axis by their localization probability. The marker proteins are shown in cyan, and the allocated proteins are in red. For each protein, the probability to belong to the outlier component is also shown in grey. The vertical dotted line in each panel indicates the protein localization prediction cutoff (localization probability threshold of 0.99). Only proteins with the localization probability above the threshold of 0.99, i.e. with the rank below the cutoff, retained their class label, whereas the rest of the proteins were labelled as ‘*unassigned*’.

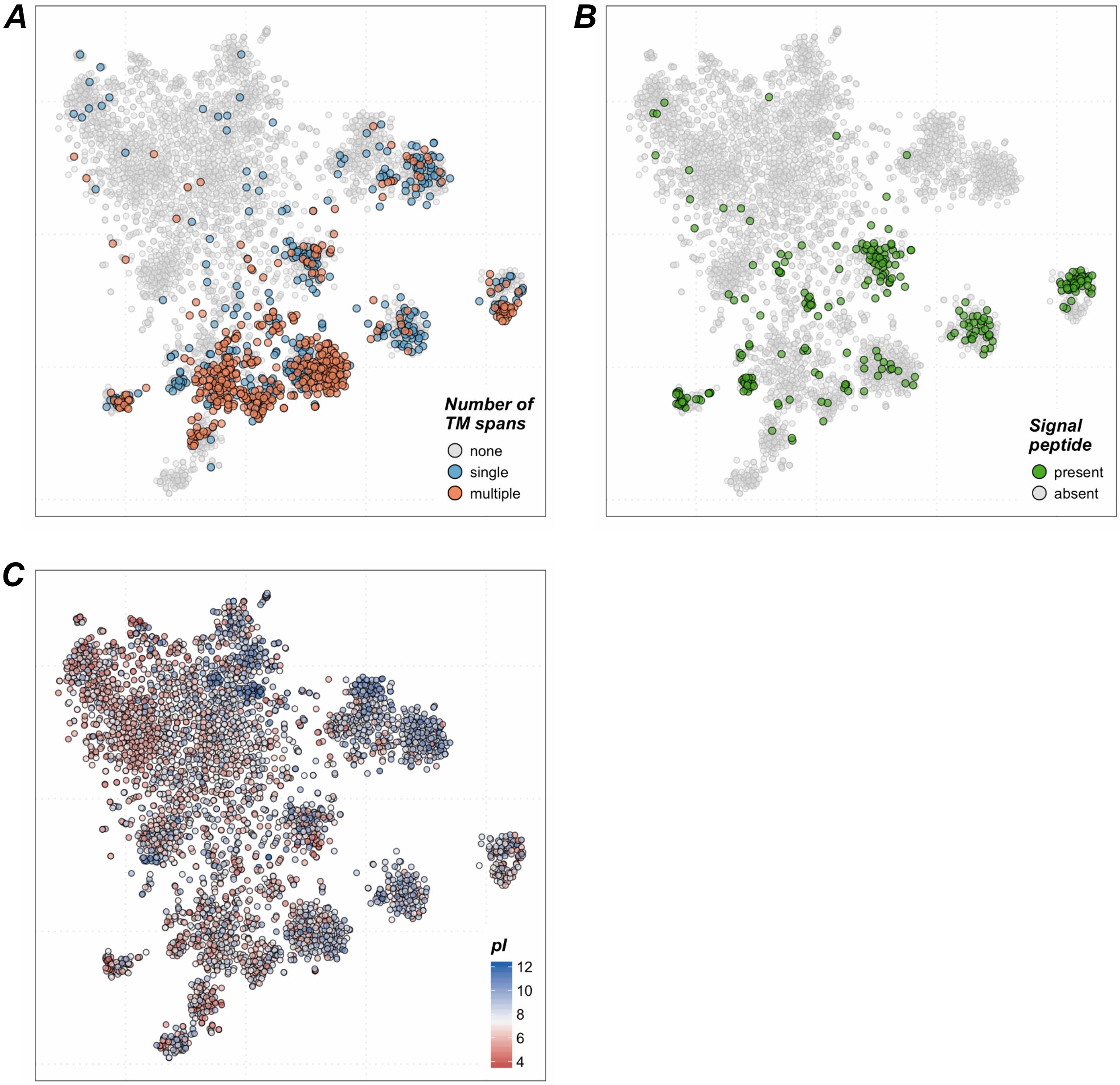

Figure S4. Distributions of select protein sequence features and properties in the spatial proteome of *T. gondii* extracellular tachyzoite, related to Figures 3, and 5.

A. A t-SNE projection of the 30plex hyperLOPIT data on 3,832 *T. gondii* proteins with monotopic (blue) and polytopic (red) integral membrane proteins highlighted. TMHMM 2.0 was used to predict transmembrane (TM) spans. The TM spans that overlapped with the signal peptide predicted by SignalP were removed.

B. Same as in ***A*** but with the proteins predicted to have a signal peptide (SignalP 5.0) highlighted in green.

C. Same as in ***A*** but showing the distribution of protein charge. Proteins are colored according to protein pI computed based on the amino acid sequence. The scale is from red for acidic proteins to blue for basic proteins with the midpoint at pI = 7.4 (colorbar in the bottom-right corner of the pane

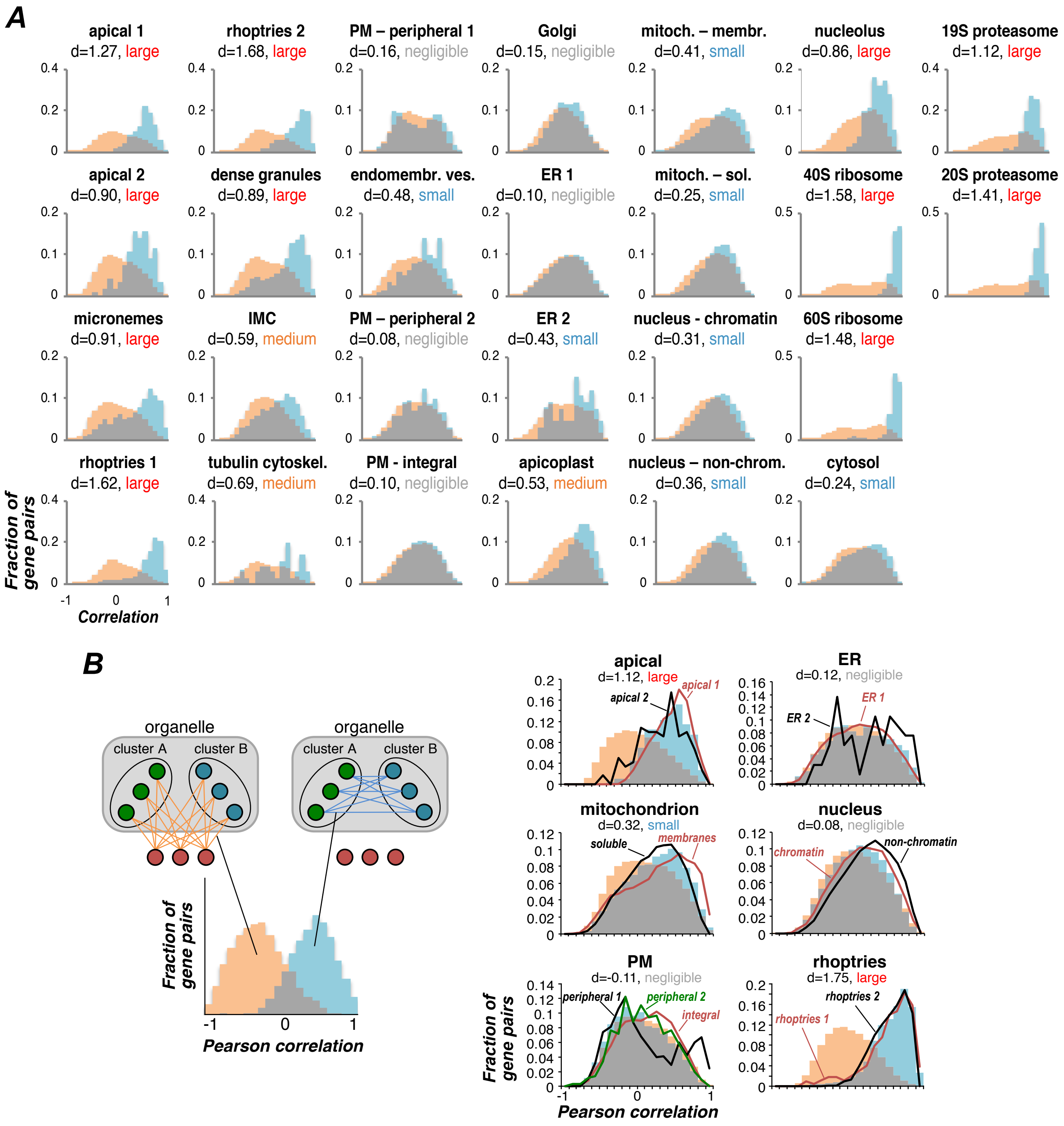

Figure S5. Gene co-expression patterns across *T. gondii* subcellular landscape, related to Figure 4.

A. Gene co-expression levels for 26 gene clusters corresponding to *T. gondii* extracellular tachyzoite subcellular compartments determined by hyperLOPIT. The co-expression levels for the genes within hyperLOPIT-defined cluster are shown as light-blue bars in histograms. The co-expression levels between the cluster members and all the genes that are not members of the cluster are shown as orange bars.

B. Gene co-expression levels within subcompartment gene clusters from select organelles. The co-expression levels between genes from hyperLOPIT-defined subcompartment clusters belonging to the same organelle (excluding co-expression levels between genes from the same cluster) are shown as light-blue bars in histograms. The co-expression levels between the cluster members from the organelle and all the genes that are not members of the clusters from the organelle are shown as orange bars. For comparison, the co-expression levels between genes from the same cluster (as in **Figure S5*A***) are shown as thin lines.

The Y-axis shows the fraction of gene pairs. The X-axis shows Pearson correlation (the range is from –1 to 1) of non-normalized quantitative transcriptomics data retrieved from ToxoDB.org. Cohen's *d* values with *effect size* descriptors are shown above each plot.

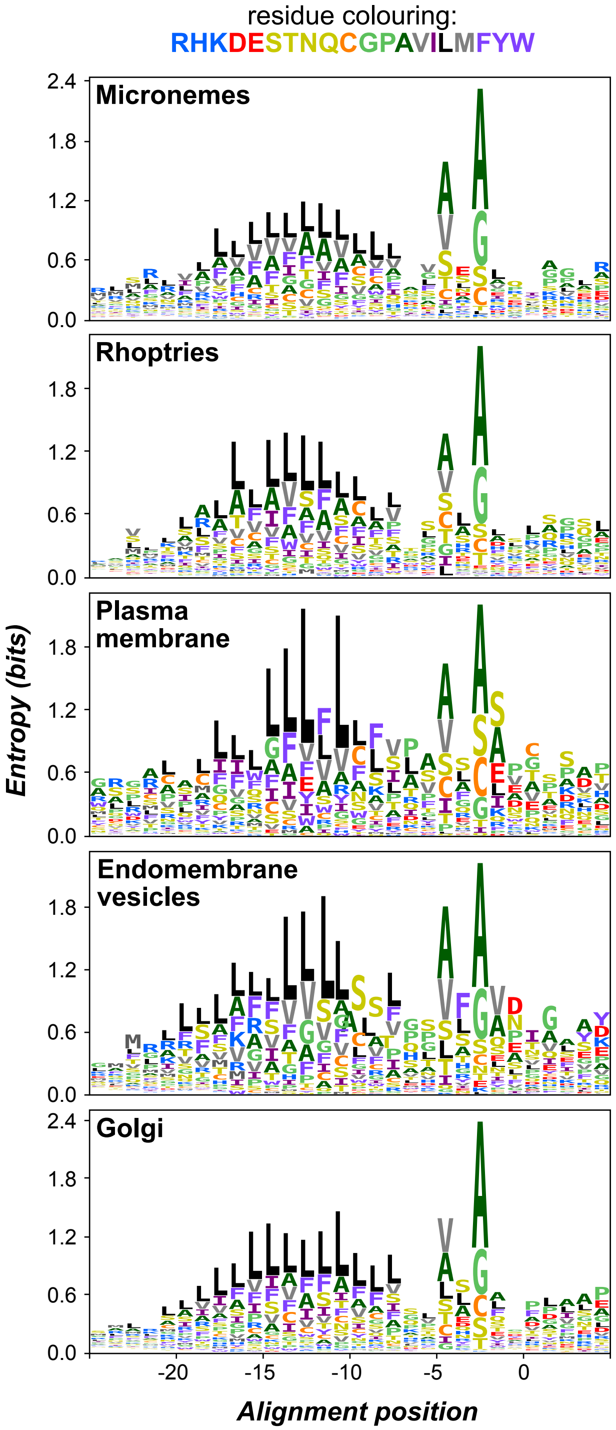

Figure S6. Logo plots of signal peptide (SP) sequences for select cohorts of *T. gondii* proteins, related to Figure 6.

The logo plots show positional abundances of amino acid residue types within and immediately downstream of the SP cleavage site. Proteins from hyperLOPIT-defined *T. gondii* compartment-specific sets and their close homologues from Apicomplexa were aligned anchored at the SP cleavage site (positon 0). Logo plots were generated after randomly sampling 1000 sequences for each data set from position-specific residue abundance probabilities calculated from dissimilarity-weighted sequences. The plots were generated in the same way as in **Figure 5*A***.

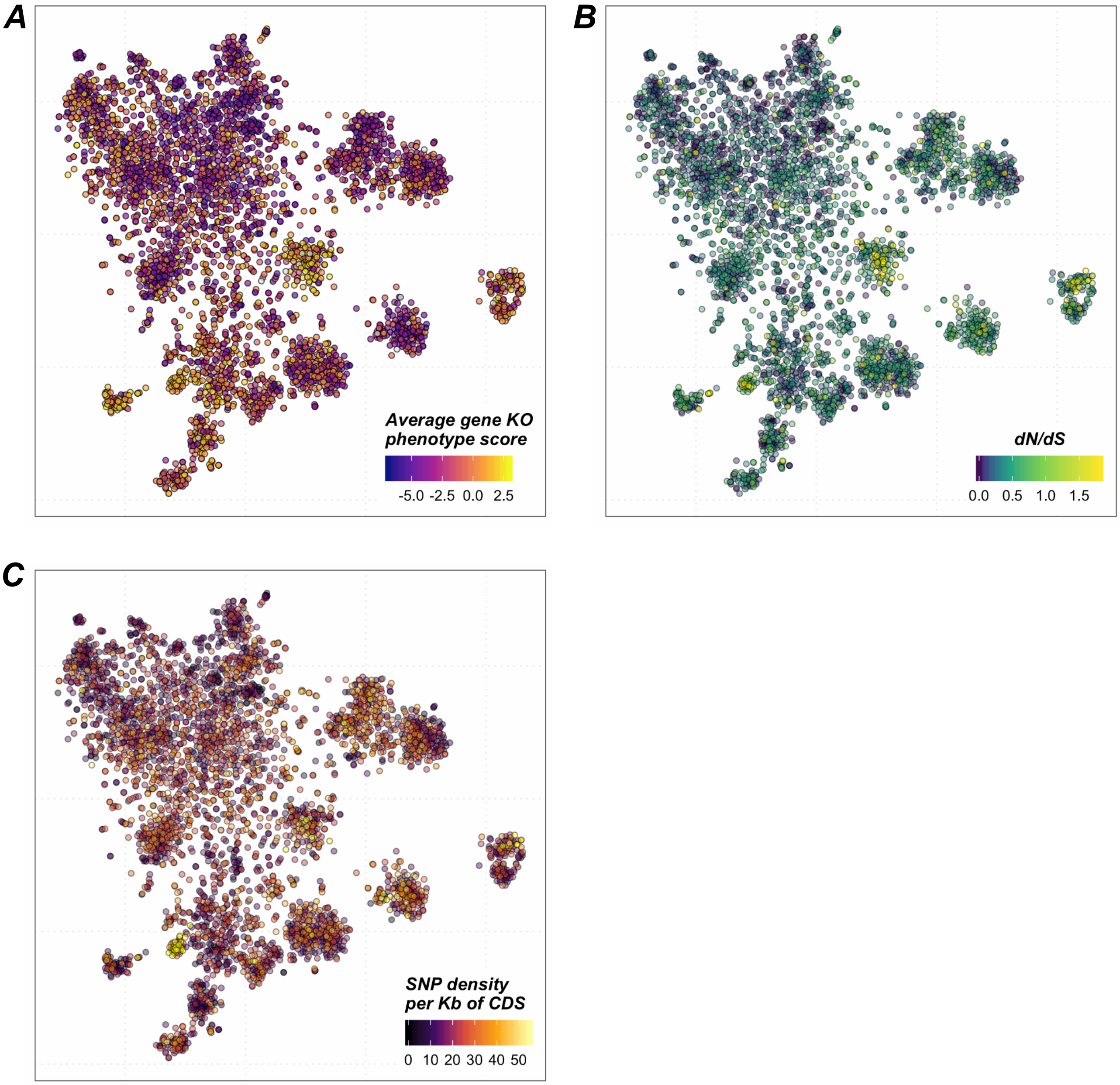

Figure S7. Mapping of the average CRISPR/Cas9-mediated gene knockout phenotype score (A), evolutionary selective pressure (B), and genetic polymorphism (C) on the 30plex hyperLOPIT t-SNE projection of *T. gondii* extracellular tachyzoite spatial proteome data, related to Figure 6.

A. Distribution of the average CRISPR/Cas9-mediated gene knockout phenotype score (Sidik et al., 2016). The range is from blue for essential genes to yellow for dispensable genes with the midpoint set at -2.4.

B. Distribution of the protein-average ratio of non-synonymous to synonymous point mutations (d_N/d_S) (Lorenzi et al., 2016). The scale is clipped at the 99-% quantile of the d_N/d_S range. Data points with extremely high (top-1%) d_N/d_S values are colored in yellow.

C. Distribution of the density of single nucleotide polymorphism (SNP) per Kb of protein-coding sequence (CDS) of genes. The SNP density data were retrieved from ToxoDB.org. As in ***A***, the scale is clipped at the 99-% quantile of the data range, with extremely high values (top-1%) shown in yellow.
