# Supplementary material for: A Comprehensive Subcellular Atlas of the Toxoplasma Proteome via hyperLOPIT Provides Spatial Context for Protein Functions
Source: Cell Host Microbe. 2020 Nov 11;28(5):752–766.e9. doi: 10.1016/j.chom.2020.09.011 (PMC7670262; doi:10.1016/j.chom.2020.09.011)
Supplement: Document S2. Article plus Supplemental Information [file mmc11.pdf]

Resource

Cell Host & Microbe

A Comprehensive Subcellular Atlas of the *Toxoplasma* Proteome via hyperLOPIT Provides Spatial Context for Protein Functions

Graphical Abstract

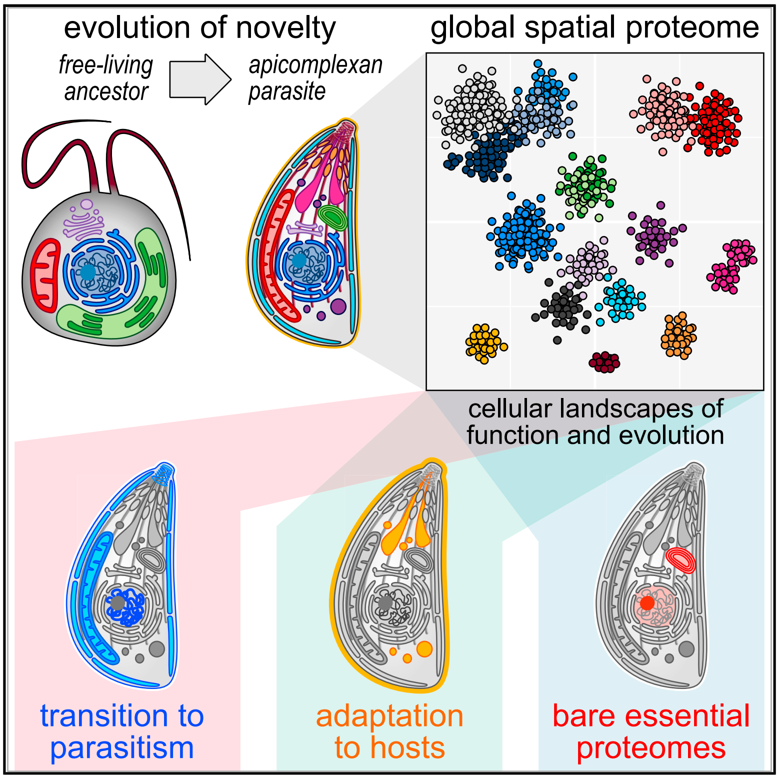

Highlights

- Using hyperLOPIT, *Toxoplasma* proteins were assigned to their cell location
- Complex proteomes associated with host interaction and adaptation are identified
- The atlas reveals sites and chronology of cell evolution of apicomplexan parasitism
- Cell spatial organization corelates with regulatory and biochemical programs

Authors

Konstantin Barylyuk, Ludek Koreny, Huiling Ke, ..., Arnab Pain, Kathryn S. Lilley, Ross F. Waller

Correspondence

kb601@cam.ac.uk (K.B.), rfw26@cam.ac.uk (R.F.W.)

In Brief

Apicomplexan proteomes are substantially specific to these parasites, and many cellular compartments are highly specialized. Using spatial proteomic methods, Barylyuk et al. simultaneously map the locations of thousands of *Toxoplasma* proteins, resolving the genomic complexity of this pathogen within the context of its cell organelles, compartments, and structures.

Barylyuk et al., 2020, Cell Host & Microbe *28*, 752–766
November 11, 2020 Crown Copyright © 2020 Published by Elsevier Inc.
https://doi.org/10.1016/j.chom.2020.09.011

CellPress

CellPress
OPEN ACCESS

Cell Host & Microbe

Resource

A Comprehensive Subcellular Atlas of the *Toxoplasma* Proteome via hyperLOPIT Provides Spatial Context for Protein Functions

Konstantin Barylyuk,[1,*] Ludek Koreny,[1] Huiling Ke,[1] Simon Butterworth,[1] Oliver M. Crook,[1,2,3] Imen Lassadi,[1] Vipul Gupta,[1] Eelco Tromer,[1] Tobias Mourier,[4] Tim J. Stevens,[5] Lisa M. Breckels,[1,2] Arnab Pain,[4,6,7] Kathryn S. Lilley,[1,2] and Ross F. Waller[1,8,*]

[1]Department of Biochemistry, University of Cambridge, Cambridge CB2 1QW, UK
[2]Milner Therapeutics Institute, Jeffrey Cheah Biomedical Centre, University of Cambridge, Cambridge CB20 0AW, UK
[3]MRC Biostatistics Unit, Cambridge Institute for Public Health, Cambridge CB2 0SR, UK
[4]Biological and Environmental Science and Engineering Division, King Abdullah University of Science and Technology, Thuwal 23955, Saudi Arabia
[5]MRC Laboratory of Molecular Biology, Cambridge CB2 0QH, UK
[6]Global Station for Zoonosis Control, Gi-CoRE, Hokkaido University, Sapporo 060-0808, Japan
[7]Nuffield Division of Clinical Laboratory Sciences (NDCLS), University of Oxford, Oxford OX3 9DU, UK
[8]Lead Contact
*Correspondence: kb601@cam.ac.uk (K.B.), rfw26@cam.ac.uk (R.F.W.)
https://doi.org/10.1016/j.chom.2020.09.011

SUMMARY

Apicomplexan parasites cause major human disease and food insecurity. They owe their considerable success to highly specialized cell compartments and structures. These adaptations drive their recognition, nondestructive penetration, and elaborate reengineering of the host's cells to promote their growth, dissemination, and the countering of host defenses. The evolution of unique apicomplexan cellular compartments is concomitant with vast proteomic novelty. Consequently, half of apicomplexan proteins are unique and uncharacterized. Here, we determine the steady-state subcellular location of thousands of proteins simultaneously within the globally prevalent apicomplexan parasite *Toxoplasma gondii*. This provides unprecedented comprehensive molecular definition of these unicellular eukaryotes and their specialized compartments, and these data reveal the spatial organizations of protein expression and function, adaptation to hosts, and the underlying evolutionary trajectories of these pathogens.

INTRODUCTION

Apicomplexa is a phylum of highly adapted unicellular eukaryotes specialized for intracellular parasitism in animals (Votýpka et al., 2017). Many apicomplexans cause devastating diseases in humans and livestock. Malaria, caused by *Plasmodium* spp., results in over 400,000 deaths and 200 million clinical cases annually, with 3.2 billion people at risk (World Health Organization, 2018). Cryptosporidiosis (*Cryptosporidium* spp.) is the second leading cause of fatal infant diarrhea affecting 800,000 annually (Kotloff et al., 2013; Striepen, 2013). Toxoplasmosis (caused by *Toxoplasma gondii*) occurs as chronic infections in ∼30% of the human population and can cause life-threatening congenital toxoplasmosis, fetal malformation and abortion, blindness, and encephalitis (Havelaar et al., 2015). Furthermore, the economic damage of disease in livestock caused by apicomplexans is estimated in billions of US dollars annually (Rashid et al., 2019). Together these pathogens have a major effect on global health and prosperity, disproportionately affecting developing world regions.

Apicomplexans are deeply divergent from better studied model eukaryotic cell systems and, as parasites, have displayed superb ingenuity for generation and specialization of cell structures and compartments. For example, a dedicated apical structure enables penetration and invasion of human and animal cells. This "apical complex" includes several highly derived secretory compartments (e.g., micronemes, rhoptries, dense granules, exonemes) for staged release of molecules required to search for, identify, penetrate, exploit, and egress from the host's cells (Figure 1A) (Kats et al., 2008; Lebrun et al., 2014; Yeoh et al., 2007). Apicomplexans have also developed novel gliding-motility structures anchored in a pellicular cytoskeleton (Frénal et al., 2017). Furthermore, modified versions of two canonical endosymbiotic compartments, the mitochondrion and a remnant of a photosynthetic plastid (apicoplast), have developed in response to the metabolic needs of obligate parasitism (Sheiner et al., 2013).

Via the secretion of complex mixtures of parasite proteins, apicomplexans also become active centers for subverting and remodeling the composition, organization, and properties of host

 Crown Copyright © 2020 Published by Elsevier Inc.
This is an open access article under the CC BY license (http://creativecommons.org/licenses/by/4.0/).

CellPress
OPEN ACCESS

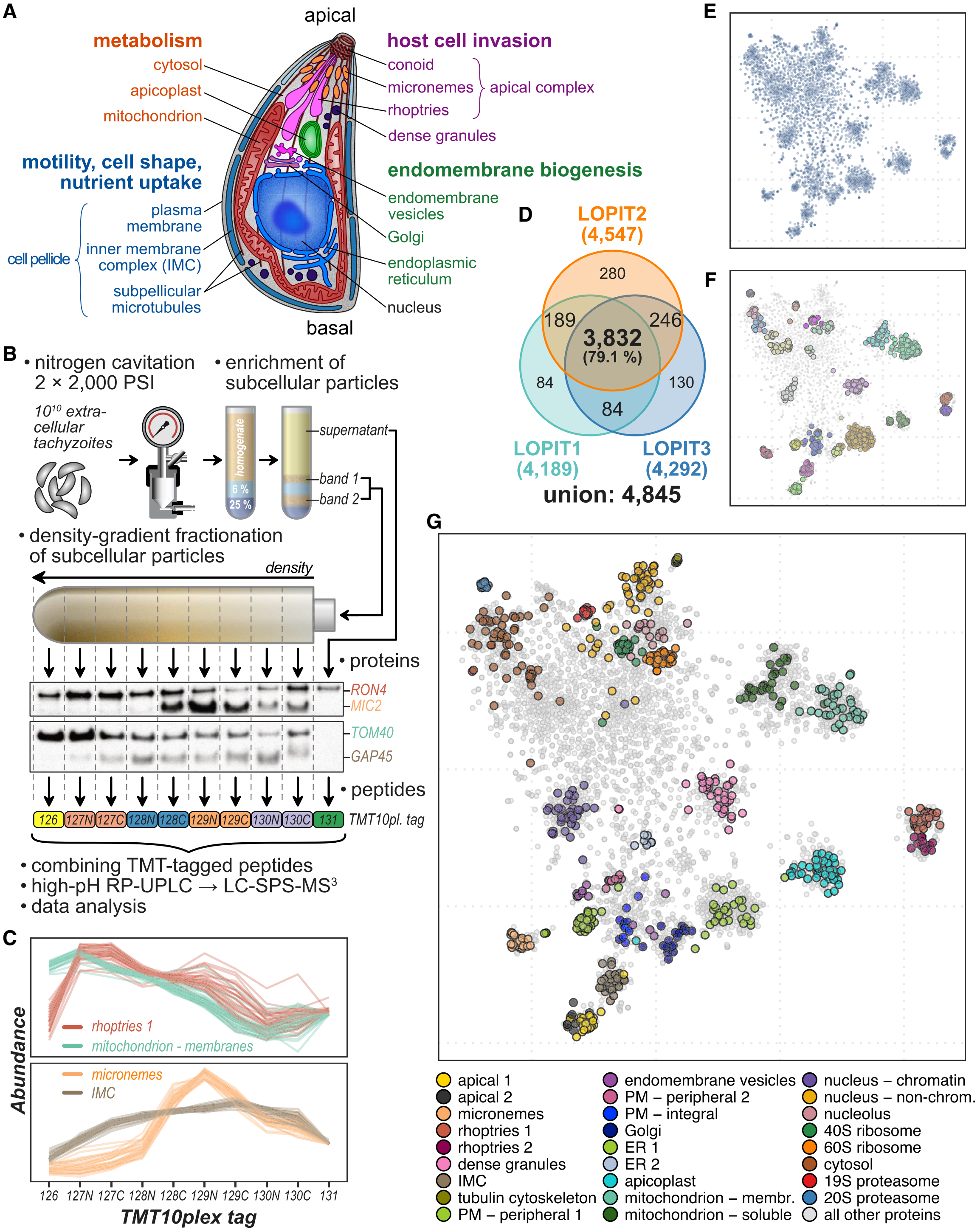

Figure 1. HyperLOPIT Reveals Organelle Protein Ensembles through Measuring Cofractionation Profiles of Proteins

(A) Schematic of *T. gondii* tachyzoite showing the main subcellular compartments and structures.

(B) Summary of hyperLOPIT workflow. Cells are mechanically disrupted, homogenate fractionated (conditions optimization by western blot, e.g., markers for rhoptries (RON4), micronemes (MIC2), mitochondria (TOM40), and IMC (GAP45)), and peptides labeled with a unique 10plex tandem mass tags for relative peptide quantitation by tandem mass spectrometry (LC-SPS-MS^3).

(C) Abundance-distribution profiles of select subcellular marker proteins measured in the LOPIT2 experiment. Note the similarity with the WB results shown in (B). See Figure S1 for concatenated profiles of all experiments (30plex).

(D) A Venn diagram showing the numbers of unique and shared proteins identified and quantified in all 10 fractions of the three hyperLOPIT experiments.

(legend continued on next page)

cells. Upon invasion, they form, and typically remain within, a "parasitophorous vacuole" decorated with parasite-secreted proteins (Cesbron-Delauw et al., 2008). Parasite-secreted proteins also target and modify existing host compartments, such as the nucleus, mitochondrion, endoplasmic reticulum (ER), cytoskeleton, and plasma membrane. In doing so, they often interfere with host control of defense and metabolism, cause reorganization of the host organelle positions and associations, change the mechanical properties of the host cell, and alter how infected cells interact with other host cells and tissues (Davies et al., 2020; Hakimi et al., 2017; Pernas et al., 2014; Soni et al., 2016). This exquisite redefinition of host cells reflects hundreds of millions of years of co-evolution with their hosts and is orchestrated by the parasite-secreted effector molecules delivered from the unique invasion machinery of these parasites. This adaptation is ongoing with contemporary changes and variability that confounds adaptive immune responses and efforts to develop effective vaccines.

The divergence and specialization of apicomplexan cell compartments limit inferences of the cell biology of these organisms that can be made from knowledge of better studied model organisms. Indeed, approximately half of apicomplexan proteins are known only as "hypotheticals" and are unique to these cells (Swapna and Parkinson, 2017). Despite decades of effort to understand the distribution of parasite proteins, typically relying on protein visualization by immunofluorescence microscopy (Woodcroft et al., 2012), the proteomes of most parasite compartments remain poorly characterized. Even the locations of proteins of predicted function based on conserved sequences in other organisms are largely untested in apicomplexans. We address this critical deficiency in apicomplexan biology, and the wider need to understand the compositional architecture of these parasites and its dynamics, by applying the spatial proteomic method hyperplexed localization of organelle proteins by isotope tagging (hyperLOPIT) (Christoforou et al., 2016; Mulvey et al., 2017) to capture the steady-state location of thousands of proteins in the apicomplexan *T. gondii*. This has provided a comprehensive understanding of the proteomic organization of an apicomplexan cell. Furthermore, these data reveal the landscapes of cellular organization, function, and evolution, including gene-expression programs, adaptative arms races with hosts, and the deeper evolutionary trajectories to parasitism.

RESULTS

Whole-Cell Biochemical Fractionation of *Toxoplasma gondii* Extracellular Tachyzoites

To determine if the steady-state subcellular locations of thousands of proteins could be simultaneously captured in apicomplexans, we adapted the hyperLOPIT method for whole-cell spatial proteomics to *Toxoplasma gondii* extracellular tachyzoites, the parasite form that is primed for host-cell invasion. The hyperLOPIT method exploits distinct abundance-distribution profiles that organelles and subcellular structures form upon biochemical fractionation such as density-gradient centrifugation. Proteins exhibiting similar distribution profiles of abundance through these fractions are assigned to distinct subcellular structures (Christoforou et al., 2016; Mulvey et al., 2017).

Optimized conditions for cell disruption and density-gradient profiles were determined using several subcellular marker proteins for western blot analysis of cell homogenates (Figure 1B). Apicomplexan infectious zoites, such as *Toxoplasma* tachyzoites, have a robust cell pellicle (Figure 1A) that is resistant to cell disruption by hypotonic lysis. Nitrogen cavitation (Wang et al., 2014) was identified as the most effective, non-heat-generating method of cell disruption. Membranous compartments and other cell particles were enriched from soluble cytosolic material by discontinuous density centrifugation of the homogenate, and this particulate material was then fractionated on continuous linear-density gradients of iodixanol, resulting in distinct enrichment profiles for a broad range of organelle markers (Figure 1B). The abundance-distribution profiles of all detectable proteins were measured by sampling nine fractions across these gradients, plus one for the cytosol material fraction, labeling the peptides of each fraction with a unique TMT10plex isobaric tag and quantifying relative peptide abundance across all fractions by mass spectrometry (Figures 1C and S1).

We performed three independent hyperLOPIT experiments, each with minor changes to cell rupturing, protein fraction preparation, and dispersal on density gradients, intending to maximize captured resolvable differences among different subcellular protein niches (Table S1). In each experiment, we identified over 4,100 proteins with quantitative information across all 10 fractions (Figure 1D); 3,832 proteins were common to all three datasets providing complete abundance-distribution profile information across 30 fractions (Figure S1; Table S2).

HyperLOPIT Assigns Thousands of Previously Uncharacterized Proteins to Subcellular Niches

The protein-fractionation data were analyzed for common abundance-distribution patterns as evidence of protein association within subcellular niches (Breckels et al., 2016; Gatto et al., 2014). To visualize the 30-dimensional data, we used the machine-learning dimensionality reduction method t-distributed stochastic neighbor embedding (t-SNE) (van der Maaten et al., 2008); t-SNE projections indicated the presence of complex structure in the data with proteins resolved into multiple, distinct, clustered sets (Figure 1E). To verify that the clusters displayed in the t-SNE projection accurately represented similarity of protein-distribution profiles and not artifacts of modeling, we analyzed the untransformed data with the unsupervised cluster-detection algorithm "hierarchical density-based spatial clustering of

(E) A 2D-projection of the 30plex quantitative proteomic data (i.e., abundance-distribution profiles) for 3,832 *T. gondii* proteins shared across three hyperLOPIT datasets. t-distributed stochastic neighbor embedding (t-SNE) was used for dimensionality reduction. Each data point represents an individual protein, and the clustering of proteins reflects the similarity of their abundance distribution profiles.

(F) Protein clusters discovered by the analysis of raw abundance-distribution profiles with HDBSCAN overlaid on the t-SNE projection. Distinct clusters are indicated by color.

(G) Mapping of 718 subcellular marker proteins on the t-SNE projection of *T. gondii* spatial proteome data.

CellPress
OPEN ACCESS

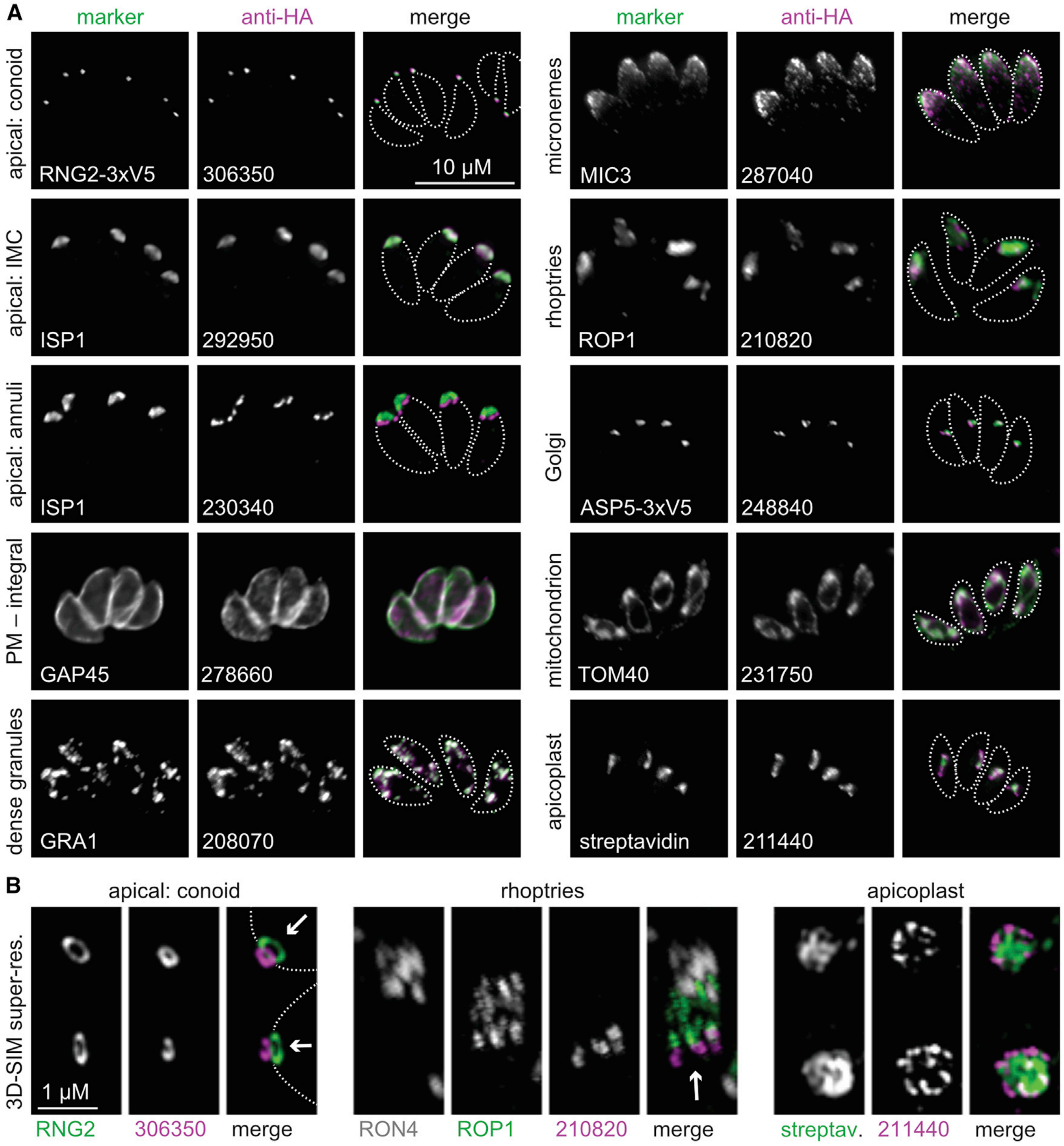

Figure 2. Validation of HyperLOPIT-Predicted Subcellular Locations

(A) Examples of uncharacterized proteins epitope tagged and detected by immunofluorescence microscopy (magenta) co-located with named marker proteins (green). Cell outlines are indicated (dashed lines). See Figure S2 for all validated proteins. Scale bar, 10 μm.

(B) Optical super-resolution (3D-SIM) images of select proteins (magenta) from (A) with subcellular marker proteins (green). Arrows indicate the cell posterior-to-anterior cell axis. Scale bar, 1 μm.

applications with noise" (HDBSCAN) (Campello et al., 2013). The clusters found in the untransformed data by HDBSCAN corresponded to the cores of many of the clusters observed in the t-SNE map, supporting the validity of these projected protein associations (Figure 1F). To assess if these clusters represent genuine biological protein assemblages, we compiled a set of 656 known marker proteins belonging to cell organelles, compartments, structures, or substructures based either on previous location studies or strong evidence of protein function (Table S3). When projected onto t-SNE maps, these markers sort according to the clusters (Figure 1G). These clusters represent all major *T. gondii* compartments or subcompartments, including many apicomplexan-specific structures, demonstrating that hyperLOPIT produced a highly resolved proteomic map of the *T. gondii* tachyzoite. The resolution of these data discerns membranous organelles (e.g., mitochondrial, micronemes, ER, Golgi); cytoskeletal elements (e.g., inner membrane complex, apical complex structures); molecular complexes (e.g., ribosome and proteasome subunits); and subcompartmental organization (e.g., outer and inner peripheral and integral plasma-membrane proteins).

To test the veracity of the hyperLOPIT clusters, 80 proteins associated with clusters representing distinct organelles or

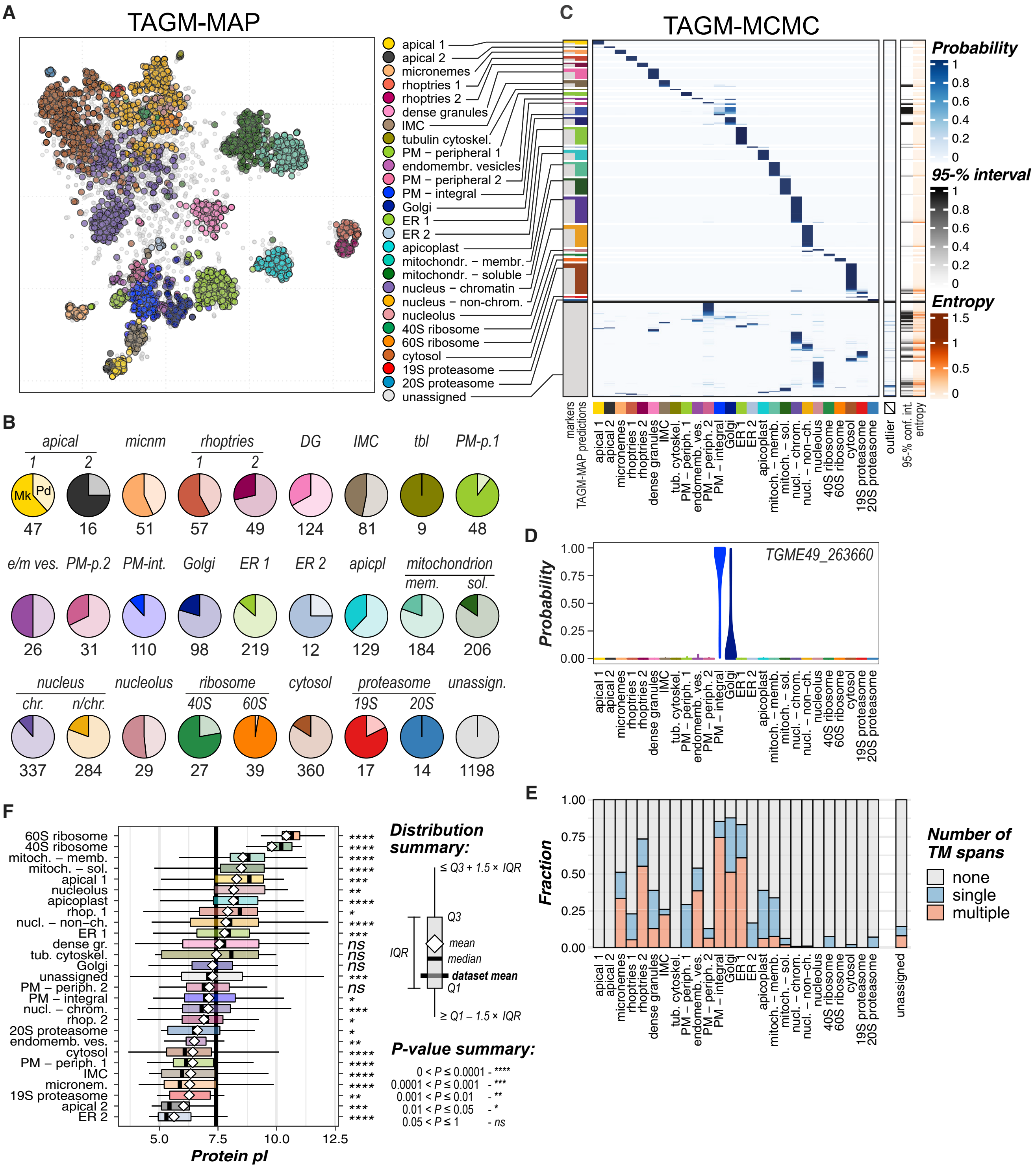

Figure 3. Protein Assignment to Known Subcellular Niches by Supervised Bayesian Classification

(A) TAGM-MAP predicted a steady-state location of proteins (99% probability) superimposed on the t-SNE projection of the 30plex hyperLOPIT data for 3,832 proteins.

(B) The number of proteins assigned to each location. Marker proteins (Mk: previously characterized proteins + verified proteins as in Figures 2 and S2) are indicated in a dark color, newly assigned protein predictions (Pd: at 99% TAGM-MAP probability) in a light color.

(C) Heatmap showing proteins ordered by the TAGM-MAP-assigned class (rows) against joint probabilities of proteins to belong to each of the 26 defined subcellular classes or the outlier component (columns) inferred by TAGM-MCMC. Colorbars on the right show the uncertainty of TAGM-MCMC localization as the 95% equitailed confidence interval of the TAGM-MCMC localization probability (in shades of gray) and the mean Shannon entropy (in shades of red).

(legend continued on next page)

CellPress
OPEN ACCESS

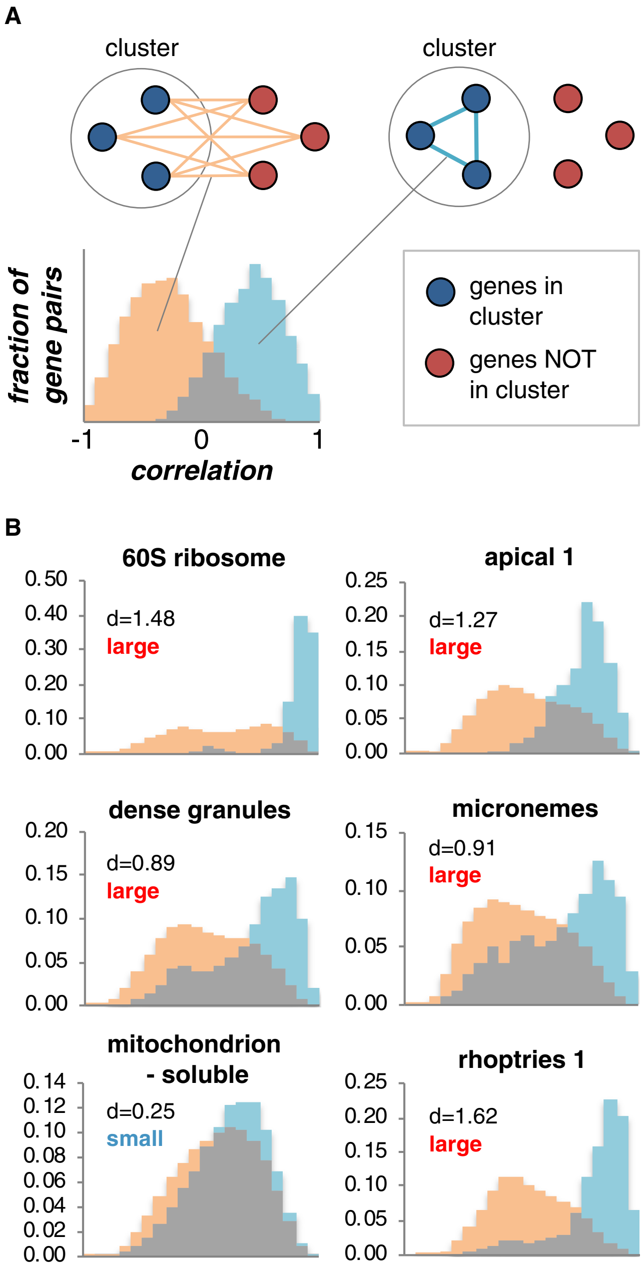

Figure 4. Correlation of Gene-Expression Patterns within Subcellular Compartments

(A) Schematic of analysis of gene co-expression according to protein location. The distribution of co-expression levels between members of a cluster (blue) is plotted against this distribution between members of the cluster and all other genes (orange).

(B) Gene co-expression levels for select hyperLOPIT clusters measured as Pearson correlations. Cohen's *d* values are shown above each chart along with *effect size* descriptors. See also Figure S5; Table S5.

subcellular structures were selected for epitope-tagging by endogenous gene fusion and immunofluorescence microscopy assays (IFAs). These proteins were previously either uncharacterized or in some cases had provisional annotation apparently in conflict with their hyperLOPIT-inferred location. Of the 80 attempted, 62 proteins could be reporter-tagged and detected by IFA, and all 62 showed subcellular location consistent with their hyperLOPIT predictions, further supporting the very high correlation of the hyperLOPIT cluster data with subcellular niches (Figures 2 and S2). The remaining 18 proteins were either recalcitrant to reporter tagging as gene fusions or were not detectable by IFA.

The resolution of protein clusters allows the prediction of the subcellular locations of all detected proteins by supervised machine-learning methods using the marker-protein distributions. The 62 newly validated proteins were added to the previous 656 markers to give 718 markers defining 26 distinct subcellular niches. We analyzed the data by a recently developed Bayesian classification method based on t-augmented Gaussian mixture models (TAGM) to probabilistically assign proteins to a set of defined classes (Crook et al., 2018, 2019). This method has the advantage of calculating a membership probability uniformly for all classes (Crook et al., 2018). This is achieved by estimates of the posterior probability of protein allocation to one of the defined subcellular classes or an outlier component which accounts for the noise in the data.

The expectation-maximization algorithm was used to compute maximum a posteriori (MAP) estimates of the TAGM model parameters from the known 718 marker proteins. Using these models, we analyzed the abundance-distribution profiles of the remaining 3,114 proteins and obtained the probability of every protein's belonging to the respective most likely subcellular class of the defined 26 and not being an outlier. We applied a uniform localization probability cut-off of 99% across all the 26 subcellular classes (Figures 3A, 3B, and S3; Table S4A). Of the 3,832 proteins measured across all three independent hyperLOPIT experiments, we assigned 1,916 proteins of previous unknown location to one of 26 subcellular niches with a localization probability above 99%. The remaining 1,198 proteins are not assigned to any location by TAGM-MAP with sufficient confidence (Figure 3B, *unassigned*).

Steady-state determination of protein locations in a population of cells overlooks the dynamic behaviors that many proteins have, including regulated location changes, trafficking intermediates, organelle contact points, and proteins with multiple locations. Occupation of multiple locations by a protein will manifest as a composite abundance-distribution profile in the hyperLOPIT data. To test if these dynamic protein behaviors can be detected, we sampled from the entire distribution of posterior location probabilities for each protein across all modeled subcellular niches using a fully Bayesian TAGM analysis employing Markov-chain Monte Carlo (MCMC) methods (Crook et al., 2018) (Figures 3C and 3D; Tables S4B and S4C). Most TAGM-

(D) A violin plot showing an example TAGM-MCMC distribution of localization probabilities across the 26 subcellular niches. The most probable location predicted by TAGM-MAP and TAGM-MCMC for this protein is *PM-integral*, but there is also a significant probability of localization to Golgi, consistent with signals seen for proteins that might cycle between multiple compartments.

(E) Fractions of monotopic and polytopic integral membrane proteins (blue and red, respectively) by subcellular class.

(F) Compartment-specific distributions of protein charge (computed pI) are shown as Tukey box plots (legend at right). The probability of class-specific means differing from the dataset average by chance is shown to the right. See also Figures S3 and S4; Tables S4 and S6A.

MAP-assigned protein locations correspond to single, high-probability location associations by TAGM-MCMC, consistent with steady-state single locations for these proteins (Figure 3C). Some TAGM-MAP-assigned compartments, however, show enrichment of proteins with probability distributions across multiple compartments by TAGM-MCMC. For instance, many integral plasma-membrane proteins show the elevated probability for the Golgi, as do endomembrane vesicle proteins (Figures 3C and 3D). By contrast, the secretory organelles of the apical complex (micronemes, rhoptries, dense granules) are dominated by single TAGM-MCMC assignments. This is consistent with a dynamic bidirectional exchange of proteins between Golgi, vesicles, and plasma membrane, whereas the proteomes of rhoptries, micronemes, and dense granules once established are static in this lifeform of *T. gondii*. Thus, TAGM-MCMC is apparently able to capture some of the dynamic properties of the *T. gondii* spatial proteome.

The TAGM-MCMC analysis also allows uncertainty quantification in the subcellular location of proteins, in particular, those proteins that are *unassigned* by TAGM-MAP (Figure 3C). In the TAGM-MAP model, the majority of these proteins have a high probability of belonging to the outlier component (Figure S3), whereas TAGM-MCMC reports high probabilities that most of them belong to a subcellular class. Many of these proteins are attributed to nuclear and cytosolic components, which could indicate their trafficking between these niches. However, we are cautious with this interpretation because of the limitations of the subcellular fractionation method used in maintaining nuclear and cytosolic integrity. The remaining TAGM-MAP *unassigned* proteins are attributed to one of the defined subcellular classes with greater uncertainty (Figure 3C), which might indicate the dynamic location behavior of these proteins.

HyperLOPIT Achieves Extensive Proteomic Resolution of Apicomplexan Cell Compartments

Interpretation of the cellular resolution of *T. gondii* achieved by hyperLOPIT requires deciphering the manner of the physical disruption and separation of organelles and subcellular structures. This, in turn, provides knowledge of proteins' and compartments' physical associations with one another and, thus, important insight into the biochemical organization of this cell.

Clear definition of distinct membrane-bound compartments (e.g., mitochondrion, apicoplast, rhoptries, micronemes, dense granules, ER) indicates that these structures were separated from one another relatively intact, and this has provided confident identification of respective proteomes. There is also evidence of some rupturing of mitochondria and rhoptries. Both organelles were resolved as two clusters with enrichment for integral-membrane proteins in one and depletion of membrane-anchored proteins in the other (Figures 3A, 3E, and S4A). This resolution is consistent with distinct abundance-distribution profiles formed by each population of proteins: (1) the membrane-attached cohort dispersing with the organelle membranes only and (2) the soluble cohort sharing a composite of the membrane profile of intact organelles and the distribution of released soluble proteins for ruptured organelles. This serendipitous distinction provided a further level of organelle proteome resolution and knowledge: proteins associated either directly or indirectly with membranous components of the organelle, and organelle-soluble proteins and complexes (Figure S4B).

The inner membrane complex (IMC) is a distinctive feature of apicomplexans, which is composed of a proteinaceous meshwork that supports flattened membranous cisternae appressed to the cytosolic face of the plasma membrane (Figure 1A). The IMC is an essential platform for motility during host invasion, maintaining cell shape and organization, and the formation of new cells during cytokinesis (Harding and Meissner, 2014). A major IMC cluster resolved separately from plasma-membrane clusters (Figures 3 and S1) indicating some level of dissociation of the IMC from the plasma membrane during cell rupturing. The tessellated membranous cisternae of the IMC line the majority of the cell body including a single conical cisterna that occupies the apical portion (~10%) of the cell—the so-called apical cap (Figure 1A). Known proteins of the apical cap and a series of small rings or "annuli" at its posterior boundary resolved separately from IMC proteins seen in the rest of the cell (Figures 2 and 3). This indicates dissociation at this boundary during cell disruption and a stronger attachment of the annuli structures to the apical cap than the posterior IMC cisternae. The apically resolved proteins also include all known *T. gondii* proteins associated with the conoid and apical polar rings, invasion-related structural components of the cell's apical extremity (Figures 2 and S2). These apical proteins resolved further as two clusters, *apical 1* and *2*, although this does not appear to represent a spatial differentiation as proteins at the apical cap and conoid occur in both (Figures 3 and S1). The basis for the hyperLOPIT resolution of these two clusters is currently unclear, but we note that protein biophysical properties also distinguish one from the other: *apical 1* enriched for basic pI, *apical 2* for acidic pI (Figures 3F and S4C). Finally, the majority of the cell's tubulin occurs in a basket of microtubules that underlie and support the IMC (Figure 1A). Tubulins, however, resolved with a select group of known subpellicular microtubule-associated proteins (MAPs) separately as a fourth cluster (Figure 3), indicating their dissociation from the proteinaceous subpellicular network of the IMC. Thus, the IMC as a definitive complex component of the apicomplexan cell pellicle resolved into four hyperLOPIT clusters of substructural associations.

The plasma-membrane proteome resolved as three biochemically distinct clusters enriched in integral-membrane proteins (*PM-integral*), peripheral proteins on the external leaflet dominated by the members of GPI-anchored surface antigen glycoprotein (SAG)-related sequence (SRS) protein family (Jung et al., 2004) (*PM-peripheral 1*), and peripheral proteins on the internal/cytosolic leaflet (*PM-peripheral 2*) (Figure 3; Table S4). ER proteins show subcompartment resolution also (Figure 3). A major class of ER proteins (*ER 1*) is enriched in integral membrane proteins. A second small group of more acidic, soluble proteins forms a distinct cluster *(ER 2)* that includes heat-shock proteins (BiP, Hsp90, and DnaK family protein), and several other proteins implicated in protein folding and processing (Figures 3E and 3F). This provides novel insight into the subcompartment organization in the ER of these parasites. The abundance-distribution profiles of *ER 2* proteins are more similar to those of the *apicoplast* rather than *ER 1* (Figure S1), suggesting some degree of association between these two. Given that most apicoplast proteins traffic through the ER, this association might reflect a role

of these proteins in folding and redox processes during sorting of proteins to the apicoplast (Biddau et al., 2018). Indeed, BiP was recently found among proteins pulled down by an apicoplast-residing thioredoxin TgATrx2 (Biddau et al., 2018).

Our implementation of the hyperLOPIT method was tailored to fractionate and resolve subcellular membranous niches, notably associated with invasion and host interaction. However, cytosolic large-protein complexes, such as the proteasome and ribosome subunits, stand out from the rest of cytosolic proteins and are, in fact, among the tightest and best resolved clusters (Figures 3 and S1). Evidence of additional structures in these regions of the hyperLOPIT maps (Figures 1E and 1F) indicates a further resolution of protein associations in these complex spaces.

Compartment Proteomes Provide Massive Expansion of Knowledge of Apicomplexan Subcellular Complexity

Of the 1,916 proteins that the hyperLOPIT could assign to known compartments with strong support, 795 (41.5%) were previously designated as "hypothetical proteins," 335 (17.5%) annotated only as conserved domain- or repeat-containing proteins, 256 (13%) annotated as generic functions, such as "transporter" or "… family protein," and for 228 (12%) their assigned function is "putative" (Gajria et al., 2008). Only 302 (16%) of these proteins showed a clearer notion of a function, typically assigned through protein similarity to conserved eukaryotic proteins, but the majority of these still lacked identified and/or experimentally validated locations. The hyperLOPIT assignments of protein location in *Toxoplasma*, therefore, provide an enormous advance in our knowledge of protein composition of subcellular compartments and niches, including those that mediate parasite-host interaction (Figure 3B).

Protein compartments that mediate parasite-host interaction is a facet of apicomplexan biology offering tremendous expansion of knowledge provided by hyperLOPIT. Three distinct secretory compartments deliver proteins either onto the parasite surface, directly into the host cytoplasm, or into membranous compartments that the parasite occupies within its host cell. Secretion from these compartments facilitates essential parasite processes: extracellular motility and host attachment (micronemes); penetration and invasion of the host cell (rhoptries); manipulation of host defenses, metabolism, and acquisition of nutrients (rhoptries and dense granules); and finally host-cell egress (micronemes in *Toxoplasma*, exonemes in *Plasmodium*) (Kafsack et al., 2009; Lebrun et al., 2014; Yeoh et al., 2007). The importance of these functions in parasite infection, virulence, and disease has focused much research attention on these compartments and their protein cargo. For each of micronemes, rhoptries, and dense granules, 29, 47, and 41 proteins, respectively, had been previously identified in *T. gondii*. HyperLOPIT identifies a further 22, 59, and 83 proteins to each of these three compartments (Figure 3B; Table S4). Of these, 22, 43, and 49 from the three respective organelles lacked apparent signal peptides that might otherwise have predicted their location to secretory organelles. We tested 15 of these signal peptide-lacking proteins and verified that all locate to their assigned organelles (Figures 2 and S2; Table S3).

The separation of rhoptries into two distinct clusters provides a new understanding of the cell biological division within this organelle: *rhoptries 1* enriched with soluble cargo; *rhoptries 2* enriched with proteins associated with membranes and hence organelle maintenance and biogenesis, even capturing maturase processes (e.g., aspartyl protease 3) of the final steps of protein sorting to rhoptries (Table S4A) (Dogga et al., 2017). Rhoptries are known to partition select proteins into the anterior tapered rhoptry "neck" from those in the posterior "bulb," and this separation correlates with timing of secretion and function: neck proteins during host penetration and bulb proteins managing the subsequent infection. While hyperLOPIT does not distinguish these populations of secreted proteins, our locating of new rhoptry proteins by microscopy did reveal further spatial organization of the rhoptries. Some proteins located exclusive to the posterior base of the bulb and others marked both the anterior and posterior rhoptry extremities (Figures 2 and S2).

The parasite surface is also a critical site of interaction with the host. The GPI-anchored SAG proteins are the best known surface molecules (Jung et al., 2004), but relatively few integral-membrane proteins in the plasma membrane that act as receptors and transporters or manage plasma-membrane properties and functions are known. The cluster of integral plasma-membrane proteins (*PM-integral*) contains 110 proteins, providing great expansion of knowledge of this proteome (Figure 3).

HyperLOPIT Resolves the Cellular Landscapes of Proteome Expression, Function, Adaptation, and Evolution within the Parasite Cell

The differential behaviors and programs of apicomplexan organelles and structures that drive protein regulation, function, adaptation, and evolution can only be resolved using comprehensive proteome samples of the different cell compartments. The hyperLOPIT spatial proteome of *Toxoplasma* provides the necessary statistical power to assess these cell properties.

Some, but Not All, Compartments Show Tight Transcriptional Regulatory Control

In *Toxoplasma*, previous efforts to identify candidate proteins for select compartments have used the correlation of transcript-abundance profiles across the cell cycle assuming that co-located proteins are co-expressed (Bai et al., 2018; Lacombe et al., 2019; Long et al., 2017; Sheiner et al., 2011). However, an objective assessment of this assumption has not been previously possible without comprehensive knowledge of the spatial distribution of the proteome. To test for compartment-correlated transcriptional control, we collated a wide range of quantitative transcriptomic data and compared within-cluster correlations of co-expression to that between a cluster and the rest of the cell proteome (Figure 4A). In several clusters, there is strong support for within-cluster co-expression (Figures 4B and S5A; Table S5). The genes for large-protein complexes show particularly strong coordinated expression: apical complex, 19S and 20S proteasome subunits, and 40S and 60S ribosomes. Membrane compartments for host invasion and interaction—micronemes, rhoptries, and dense granules—also show strong coordinated expression, as do the apicoplast and the IMC, although with less support. Other compartment-wide proteomes showed either smaller or no evidence of coordinated expression (e.g., soluble mitochondrion proteins). Where hyperLOPIT has resolved the proteomes of organelle subcompartments (e.g., *rhoptries 1* and *2*), there is no evidence of a difference in gene-

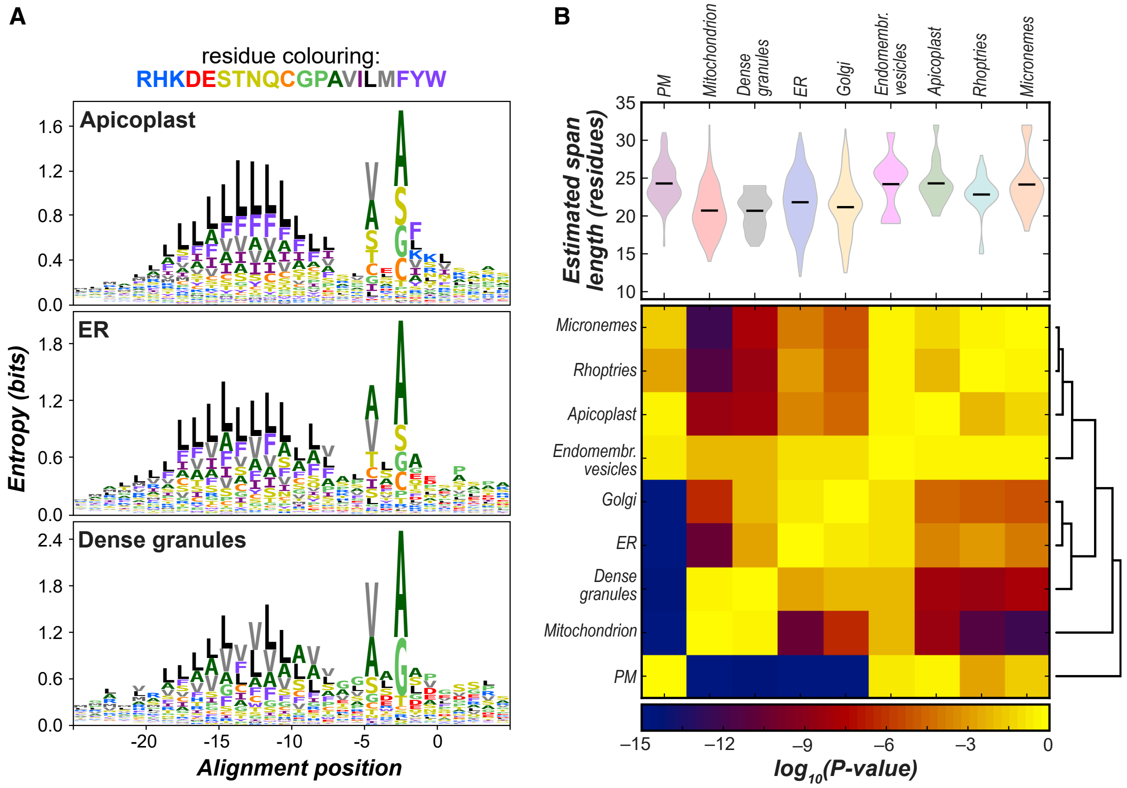

Figure 5. Distinction of Properties of Apicomplexan Signal Peptide and Transmembrane Domain Sequences According to Subcellular Compartment

(A) Differences in relative positional abundances of amino acids for signal peptide (SP) sequences of proteins from apicomplexan endomembrane compartments shown as logo plots anchored on the cleavage site (position 0). See also Figure S6; Tables S7A and S7B. Amino acids are colored by physicochemical properties.
(B) Distributions of apicomplexan transmembrane (TM) span length for single-span proteins of different compartments. The length distributions (violin plots) were compared pairwise by the Mann-Whitney U test, and the resulting p values (heatmap) were used to cluster membrane type. See also Table S7C.

expression patterns between these subproteomes (Figure S5B; Table S5).

Subcellular Proteomes Reveal Biophysical and Functional Partitioning of the Cell

Protein properties are adapted to the environments and processes in which they operate. Proteomic data from subcellular niches, therefore, can report on the biochemical conditions of these microenvironments across cell compartments and programs. Protein pI values often reflect the pH of their local environment, and clear differences are seen in average pI values of proteins in the different subcellular niches (Figures 3F and S4C; Table S6A). This includes the stepwise acidification through the secretory pathway: from ER to Golgi to endomembrane vesicles. It also reveals an apparently basic pH of the apicoplast, a property of this organelle that was previously not known and which could indicate a role of pH in protein import.

Our data also report differences in transmembrane-trafficking programs and membrane properties throughout the cell. The Sec61 complex is a common entry point for protein into the endomembrane system from which they are sorted to multiple destinations, many central to host interactions. Co-translational ER import is mediated by interactions of the ER-import machinery with cleavable N-terminal signal peptides. Comparison of signal peptides from *T. gondii* and their apicomplexan orthologues for different endomembrane niches reveal statistically significant compositional differences between protein groups destined to different locations (Figure 5A and S6; Table S7B). Signal peptide sequences are known to modulate the kinetics of Sec61 translocation and signal cleavage, both of which affect folding and chaperone recruitment, and in cases of ER stress, signal peptides can even selectively reroute proteins for cytosolic destruction (Kang et al., 2006; Snapp et al., 2017; Sun et al., 2020). Our data imply that these or similar processes provide a level of sorting selection for apicomplexan secretory compartments at these early stages of protein synthesis.

Membrane-spanning proteins interact with the lipid bilayers that they are embedded into. Analysis of the distribution of lengths of apicomplexan single-span protein transmembrane (TM) domains shows clear differences between compartments (Figure 5B). These differences most likely reflect lipid compositional difference across the cell, and experimental evidence on select proteins has shown that TM span length can govern protein-sorting events in apicomplexans (Karsten et al., 2004). We show that microneme proteins share long TM spans with those of the plasma membrane consistent with this being the destination of microneme proteins once secreted. By contrast, dense granule proteins do not follow the increase in TM span length seen from early to late parts of the secretory pathway

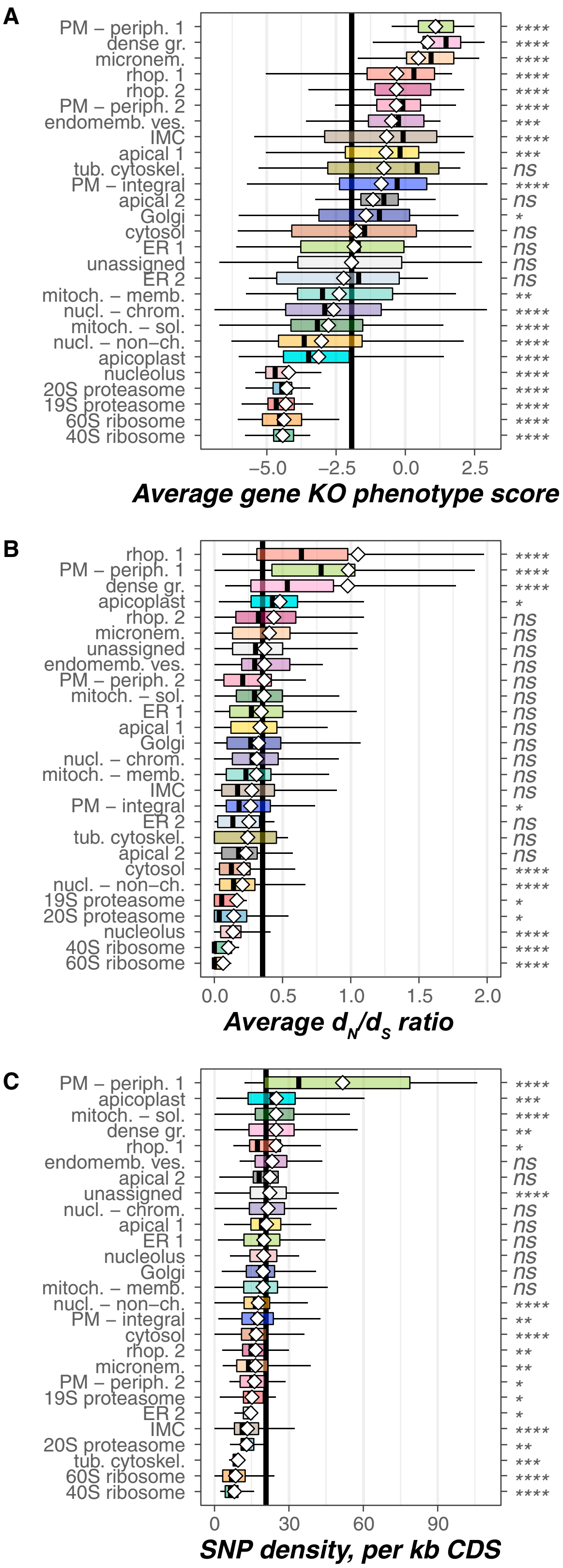

Figure 6. *T. gondii* Subcellular Compartments Show Distinct Distributions of the Functional Redundancy of the Proteomes, Selection Pressure, and Genetic Polymorphism

(A) Compartment-specific distribution of protein functional redundancy expressed as the average gene knockout (KO) phenotype score quantifying the contribution of each *T. gondii* gene to the parasite fitness during *in vitro* culture (a negative score indicates relatively indispensable genes; a positive score indicates dispensable genes).

(B) Compartment-specific distributions of evolutionary selection pressures expressed as the protein-average ratio of nonsynonymous and synonymous mutation rates (d_N/d_S ratio).

(C) Compartment-specific distributions of genetic polymorphism expressed as the density of SNP per kilobase of gene coding sequence (CDS).

Compartment-specific distributions are shown as Tukey box plots as for Figure 3F.

See also Figure S7; Tables S6B–S6D.

(Sharpe et al., 2010). Dense granule proteins must avoid insertion into the parasite plasma membrane post-secretion, and their trend for shorter TM spans might contribute to their onward trajectory into the host.

The relative redundancy of proteomes across the subcellular landscape was also assessed using our extensive representation of compartment proteomes. Data from a genome-wide CRISPR-Cas9 knockout screen in *T. gondii* were employed where phenotype was measured during *in vitro* tachyzoite propagation (Sidik et al., 2016). Combining this genetic screen with unambiguous evidence of protein expression in tachyzoites enables the uneven compartment distribution of relatively dispensable versus indispensable proteins to be seen (Figures 6A and S7A; Table S6D). The plasma membrane (including *PM-integral*), dense granules, micronemes, rhoptries, and the IMC show the largest bias for dispensable proteins in these conditions (Figure 6A). These compartments, therefore, apparently do not follow the otherwise common trend of parasite gene loss and complexity minimalization. By contrast, other compartments such as the apicoplast show a paucity of dispensable proteins (Figure 6A). Thus, despite this organelle being a remnant of a former photosynthetic lifestyle and its early interpretation as "evolutionary baggage," it is now clear that it has become a highly reduced organelle supported by a bare essential proteome.

Heterogeneous Compartment Host-Adaptive Responses

Parasites of humans and animals operate under enormous selective pressures to successfully exploit available hosts—all under constant surveillance and attack by the host immune system. As a zoonotic infectious agent, *T. gondii* is also adapted to exploiting a variety of different warm-blooded organisms. The strength and nature of selective pressures on a protein is evident by the ratio of rates of non-synonymous (d_N) to synonymous (d_S) point mutations for a gene, and the distribution of gene d_N/d_S values informs on the within-cell distribution of these pressures and parasite responses across compartments. Gene single nucleotide polymorphism (SNP) properties were analyzed across the subcellular compartments from population data for 62 *T. gondii* geographical isolates (Figures 6B, 6C, S7B, and S7C; Tables S6B and S6C) (Lorenzi et al., 2016). Compartments with highly positive-skewed d_N/d_S distributions are those of the external peripheral plasma-membrane proteins, the soluble content of rhoptries, and the dense granules (Figure 6B). This implies

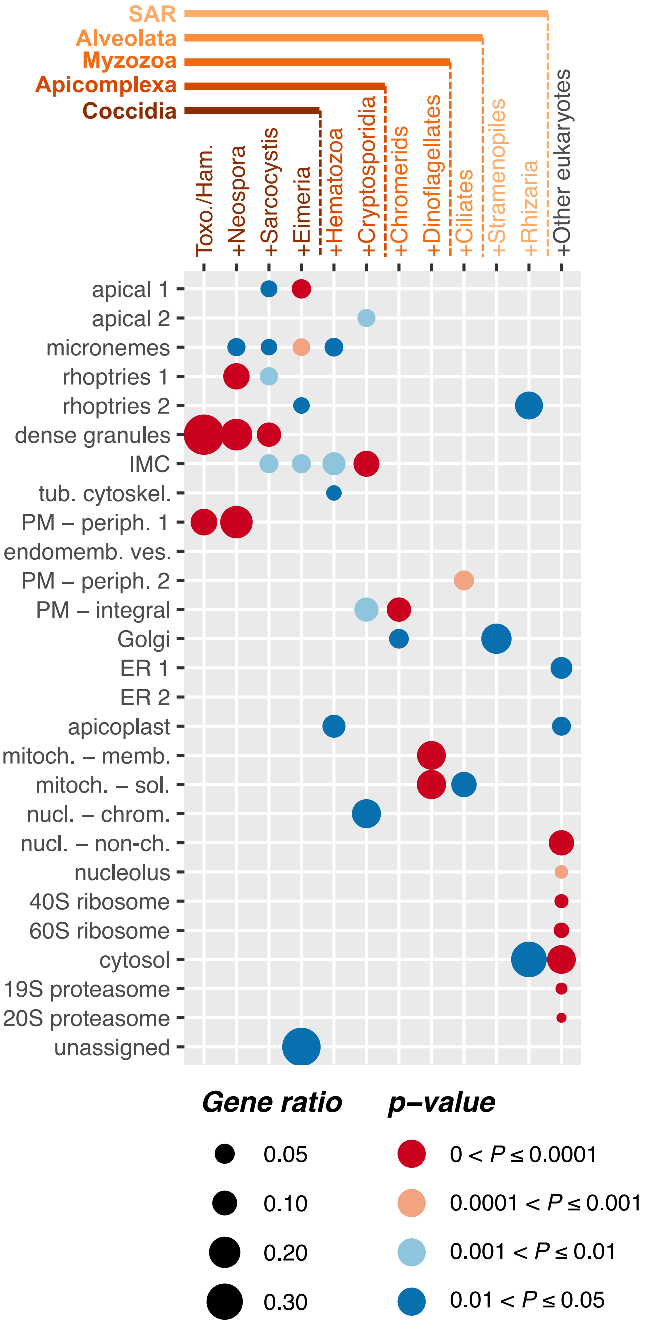

Figure 7. *T. gondii* Subcellular Compartment Proteomes Reveal the Tempo of Compartment Evolution Over Evolutionary Time

A dot plot showing the distribution of significant enrichments for new protein orthologues at twelve phylogenetic distance levels within hyperLOPIT-defined apicomplexan compartment classes. p values (colors) calculated by under-representation hypergeometric test and scaled according to the gene ratio (fraction of novel proteins in a compartment against all novel proteins at a given phylogenetic distance level). Toxo./Ham., *Toxoplasma/Hammondia*; SAR, stramenopiles/Alveolata/Rhizaria.

See also Tables S8A–S8F.

strong positive selection for change but also a high capacity of proteins in these niches to tolerate changes. Such proteins within these compartments are likely at the frontline of host-pathogen interaction and adaptation. In stark contrast to the high rate of change of the peripheral external plasma-membrane proteins is that of the integral plasma-membrane proteins, which are biased for purifying selection (low d_N/d_S) (Figure 6B). These differences reveal the tension between exposure to host-immune factors and the maintenance of plasma-membrane function. Other cell niches under purifying selection are those for central cellular function: ribosomes, cytosol, nonchromatin nuclear proteins, nucleolus, and proteasome (Figure 6B).

SNP density within coding sequences also responds to compartment evolution, and for many compartments SNP density correlates with d_N/d_S (e.g., both high for plasma-membrane peripheral proteins, low for ribosomes) (Figure 6C). An unexpected mutation behavior, however, is observed with the mitochondrial soluble proteins that show significant enrichment for higher than average SNP densities (Figure 6C), but no increase in d_N/d_S (Figure 6B). This enrichment for synonymous, or "silent," mutations indicates selection for codon-usage changes across strains. This likely has implications for translation-efficiency differences and metabolic flux control in this important metabolic compartment. A similar bias for SNP density is seen in the apicoplast also, although here some selection for protein sequence change is also seen (Figures 6B and 6C). Thus, modulation of metabolic control might be an important driver of host tissue and/or taxon preference or even virulence across parasite populations.

Compartment-Specific Evolutionary Trajectories to Parasitism in Apicomplexa

A resolved apicomplexan spatial proteome also allows the broader evolution of apicomplexan parasites to be assessed. We asked the question, When in the evolution of these parasites did different cell compartments and functions display the greatest rates of innovation? We surveyed the distribution of new protein orthologues across cell compartments over phylogenetic distance (Figure 7; Tables S8A–S8F). These data show that different cell compartments display very different rates of evolutionary protein innovation. At the most ancient level of the last eukaryotic common ancestor (LECA), as expected, orthologues are enriched in core cellular compartments, including the cytosol and complexes for protein expression, sorting, and turnover (Figure 7). By contrast, the compartments most enriched for recent, coccidian-specific orthologues include the dense granules, rhoptry soluble fraction, micronemes, apex, and peripheral surface proteins—all components of the cell that define the interaction with its hosts. Dense granules show the greatest novelty and are apparently most instrumental to the recent evolution in *Toxoplasma* and its close relatives. Other cellular locations show earlier, apicomplexan-specific accelerated evolution, which has likely been important to the adaptation of apicomplexans as parasites: the IMC, which is key to parasite motility, host contact, and invasion, and the *nucleus-chromatin* cluster, which is consistent with the evolution of novel gene-regulatory networks shared by parasites (Woo et al., 2015). Chromerids are apicomplexans' closest photosynthetic relatives, and they also live in association with animal communities (Janouškovec et al., 2013). Innovation in the integral plasma-membrane proteome, notably enriched in membrane transporters, is seen in these group's common ancestors that might indicate the beginning of a molecular exchange with animal partners. An even

CellPress
OPEN ACCESS

deeper, rapid change in the mitochondrion is evident prior to the divergence of apicomplexans from dinoflagellate algae, and this is consistent with many known peculiarities of this essential metabolic organelle shared by both groups (Danne et al., 2013; Waller and Jackson, 2009). And finally, in the common ancestor of the Alveolata, the group that includes ciliates and is defined by the pellicle organization of subplasma membrane alveolae (IMC), enrichment for new inner-leaflet peripheral plasma-membrane proteins is seen. These proteins include several Ca^{2+}- and cGMP-receptive molecules (e.g., calcium-dependent protein kinase 3, protein kinase G) of the signaling cascades that are central to apicomplexan invasion and host egress events. This is the first biochemical evidence for the common coupling of this cell ultrastructure with this critical function so early in apicomplexan evolution. Collectively, these data provide an unprecedented view of the evolutionary chronology of apicomplexan cells and their trajectory to parasitism.

DISCUSSION

The application of hyperLOPIT to the *T. gondii* extracellular tachyzoite provides a comprehensive high-resolution spatial proteomic map of an apicomplexan cell including both generic and specific compartments. These data uncover a myriad of layers of insight into the biochemical, functional, and evolutionary organization of these major human pathogens. Overall, we identified and quantified 3,832 proteins and assigned 2,634 proteins to 26 distinct subcellular niches with 99% Bayesian posterior probability. These include virtually all known *Toxoplasma* cell compartments including those specific to the majority of apicomplexan parasites. The immediate outcome of our study is a massive expansion of known organelle proteomes. For invasion organelles—micronemes, rhoptries, and dense granules—this provides tremendous new knowledge of the complexity of effector repertoires secreted into the host upon *T. gondii* invasion, growth, and egress. We also capture the proteins involved in the biogenesis and maintenance of these organelles. Major new elements of the proteomes of the apical subdomain of the IMC and the apical cytoskeletal structures including the conoid have also been discovered, and this has led to wider known conservation of these structures in apicomplexans (Koreny et al., 2020). Since these subcellular niches are phylogenetically restricted to apicomplexans and their close relatives, the empirical definition of their biochemistry is essential to understand their functions. Furthermore, with hundreds of proteins assigned to the mitochondrion and apicoplast, the metabolic capacity and activities of these otherwise enigmatic endosymbiotic organelles in parasites can now be addressed in far greater depth.

Because hyperLOPIT is independent from inferences of protein function and/or location from conserved domains, sequence motif prediction, or orthologues in other organisms, it does not suffer from potential pitfalls of these approaches. For example, ToxoDB: TGME49_310290 is annotated as a "regulator of chromosome condensation (RCC1) repeat-containing protein" based on sequence similarity, suggesting a nuclear location. This protein, however, was assigned to the *mitochondrion-soluble* class by hyperLOPIT and validated by microscopy (Figure S2). Similarly, the annotated "rhoptry kinase family proteins," ROPs, suggest rhoptry localization, as is the case for many such proteins (e.g., ROP11, 20, 24, 26) (Lebrun et al., 2014). However, several "ROP" proteins (ROP32-35: ToxoDB: TGME49_270920, ToxoDB: TGME49_201130, ToxoDB: TGME49_240090, and ToxoDB: TGME49_304740) were attributed to dense granules by hyperLOPIT and recently confirmed experimentally for ROP34 and ROP35 (Beraki et al., 2019). Moreover, proteins can relocate between compartments over evolutionary time, further confounding orthology-based inferences. For example, apicomplexans have expanded a family of mitochondria-targeted RNA-binding proteins (RAPs) with *Toxoplasma* encoding 16 such RAP-domain proteins (Lee and Hong, 2004; Woo et al., 2015). Most were assigned to the *mitochondrion-soluble* class by hyperLOPIT as expected. One RAP protein (ToxoDB: TGME49_211890), however, which is a product of a Sarcocystidae-specific gene duplication, was assigned to the apicoplast and this location was also verified (Figure S2). While the organellar function of RAP proteins awaits discovery, this relocation of a mitochondrial protein to the apicoplast indicates an evolutionary transfer of function between cell compartments. Such transfers might also account for "ROPs" being dispersed in rhoptries and dense granules as parasite-host interactions continue to evolve.

There are several reasons why a fraction of proteins has not been confidently assigned to a subcellular compartment. First, TAGM is unable to model unknown subcellular niches or those that lack sufficient known proteins to serve as markers. Proteins that occur in such cellular niches will be erroneously classified to one of the known clusters, typically with lower probabilities, or the outlier component (Crook et al., 2018). Second, for very low-abundant proteins the measured abundance-distribution profiles may suffer distortion by the noise of low signals. We note, however, that many proteins even below detectable levels by western blot or IFA were still reproducibly quantified and assigned by hyperLOPIT. Most importantly, the hyperLOPIT method reports protein steady-state locations; however, some proteins distribute between more than one organelle and cannot be unambiguously assigned to any class (Crook et al., 2018; Thul et al., 2017). We found that the greatest proportion of uncertain assignments were between cytosolic and nuclear protein clusters, as well as between *Golgi*, *endomembrane vesicles*, *PM-integral*, and *PM-peripheral 2* classes. The former is probably because many proteins shuttle between the cytosol and the nucleus, and also that the cell disruption and fractionation method may have disrupted nuclear integrity. The latter likely reflects the intrinsic heterogeneity and dynamic nature of endomembrane compartments of the secretory pathway.

This comprehensive and unbiased spatial proteome nevertheless opens up opportunities for discovering previously uncharacterized subcellular niches. The t-SNE projections indicate a considerable structure in regions of these maps beyond that accessible to TAGM analysis for the lack of known markers for these clusters. The unsupervised analysis of the data (HDBSCAN) supports further genuine protein associations here. This provides routes to discover previously unrecognized cellular organization.

The pursuit of subcellular proteomes in apicomplexans has often focused on identifying molecular machinery that can elucidate the functions and processes of cell compartments. Indeed, these hyperLOPIT data provide innumerable opportunities now for these important pursuits. Moreover, in combination with

genetic screens, made more accessible with CRISPR-Cas9 and modern DNA-sequencing methods, hyperLOPIT data provide a means to interpret the outcomes of these screens in a subcellular context. However, there is now also tremendous opportunity to understand broader processes of cell function, adaptation, and evolution, by using these objective comprehensive samples of the compositional organization of the cell in combination with other systems-level data. For example, gene-expression analysis reveals tight expression programs for some large molecular complexes and for invasion-related structures that likely contribute to the ordered assembly of these apicomplexan apparatuses central to pathogenicity. Further, a cell-wide view of the distribution of selective pressures and their responses, manifesting as population-level skews in gene d_N/d_S and SNP-frequencies across compartments, shows how nonuniformly contemporary adaptation occurs in these parasites. Proteins changing most rapidly are enriched in rhoptries, dense granules, and at the parasite surface, and these likely identify molecular processes most relevant to the host-parasite arms race of attack and defense. But equally importantly, these data reveal proteins within such compartments that do not change and which might present stable targets for therapeutic strategies against these parasites' core processes. Finally, deeper evolutionary questions can be asked about the relative chronology and tempo of innovation in the different cell compartments over evolutionary time. These reveal a sequence of innovation, from early to late, in functional development for intracellular signaling cascades, metabolism, the extracellular interface, genetic networks, motility, invasion, and, finally, host remodeling; our analysis also identifies the proteins responsible for these key events (Table S8F). Combating apicomplexans as pathogens requires understanding the fundamentals of their evolution as parasites, as well as the nuances of modern adaptation and molecular function. These high-resolution spatial proteomic data for apicomplexans offer a new era of discovery and advancement of our understanding of and approaches to tackling these critical human pathogens.

STAR★METHODS

Detailed methods are provided in the online version of this paper and include the following:

- KEY RESOURCES TABLE
- RESOURCE AVAILABILITY
  - Lead Contact
  - Materials Availability
  - Data and Code Availability
- EXPERIMENTAL MODEL AND SUBJECT DETAILS
- METHOD DETAILS
  - Generation of Transgenic *T. gondii*
  - Immunofluorescence Microscopy and Immunoblotting
  - Sample Preparation for hyperLOPIT
  - HyperLOPIT Subcellular Fractionation
  - Proteomic Sample Generation
  - Chromatographic Prefractionation of Peptides
  - LC-MS Analysis of Peptides
- QUANTIFICATION AND STATISTICAL ANALYSIS
  - Raw LC-MS Data Processing and Quantification
  - Location Prediction by Machine Learning Methods
  - Prediction of Signal Peptides and TM Spans
  - Distribution of Gene and Protein Characteristics
  - Analysis of Gene Co-expression
  - Gene Orthology Analysis
  - Properties of Signal Peptides and TM Spans
  - Protein Sequence Logo Plots

SUPPLEMENTAL INFORMATION

Supplemental Information can be found online at https://doi.org/10.1016/j.chom.2020.09.011.

ACKNOWLEDGMENTS

This work was supported by the Medical Research Council, United Kingdom, MR/M011690/1 to R.F.W.; King Abdullah University of Science and Technology (KAUST), Saudi Arabia, OSR-2015-CRG4-2610 to A.P., R.F.W., and K.S.L.; Wellcome Trust, United Kingdom, Investigator award 214298/Z/18/Z to R.F.W.; an Isaac Newton Trust-Leverhulme, Early Career Fellowship ECF-2015-562 to K.B; and KAUST faculty baseline funding (BAS/1/1020-01-01) to A.P. Mass spectrometry data were acquired by Mike Deery at the Cambridge Centre of Proteomics, and we thank Laurent Gatto for useful discussions.

AUTHOR CONTRIBUTIONS

K.B., K.S.L., and R.F.W. conceived this study. K.B. performed and analyzed the hyperLOPIT experiments, with O.M.C. and L.M.B. contributing to the design and implementation of the analyses. L.K., H.K., S.B., and I.L. performed gene-tagging location validations; V.G. and E.T., the orthology analysis; T.M. and A.P., the gene-expression analysis; and T.J.S., the signal peptide and transmembrane domain analysis. K.B. and R.F.W. wrote the manuscript, which was read and approved by all authors.

DECLARATION OF INTERESTS

The authors declare no competing interests.

Received: May 11, 2020
Revised: July 30, 2020
Accepted: September 15, 2020
Published: October 13, 2020

REFERENCES

Almagro Armenteros, J.J., Tsirigos, K.D., Sønderby, C.K., Petersen, T.N., Winther, O., Brunak, S., von Heijne, G., and Nielsen, H. (2019). SignalP 5.0 improves signal peptide predictions using deep neural networks. Nat. Biotechnol. *37*, 420–423.

Aurrecoechea, C., Brestelli, J., Brunk, B.P., Dommer, J., Fischer, S., Gajria, B., Gao, X., Gingle, A., Grant, G., Harb, O.S., et al. (2009). PlasmoDB: a functional genomic database for malaria parasites. Nucleic Acids Res. *37*, D539–D543.

Aurrecoechea, C., Brestelli, J., Brunk, B.P., Fischer, S., Gajria, B., Gao, X., Gingle, A., Grant, G., Harb, O.S., Heiges, M., et al. (2010). EuPathDB: a portal to eukaryotic pathogen databases. Nucleic Acids Res. *38*, D415–D419.

Bai, M.J., Wang, J.L., Elsheikha, H.M., Liang, Q.L., Chen, K., Nie, L.B., and Zhu, X.Q. (2018). Functional characterization of dense granule proteins in Toxoplasma gondii RH strain using CRISPR-Cas9 system. Front. Cell. Infect. Microbiol. *8*, 300.

Benjamini, Y., and Hochberg, Y. (1995). Controlling the false discovery rate: a practical and powerful approach to multiple testing. J. R. Stat. Soc. B *57*, 289–300.

Beraki, T., Hu, X., Broncel, M., Young, J.C., O'Shaughnessy, W.J., Borek, D., Treeck, M., and Reese, M.L. (2019). Divergent kinase regulates membrane ultrastructure of the Toxoplasma parasitophorous vacuole. Proc. Natl. Acad. Sci. USA *116*, 6361–6370.

CellPress
OPEN ACCESS

Biddau, M., Bouchut, A., Major, J., Saveria, T., Tottey, J., Oka, O., Van-Lith, M., Jennings, K.E., Ovciarikova, J., DeRocher, A., et al. (2018). Two essential thioredoxins mediate apicoplast biogenesis, protein import, and gene expression in Toxoplasma gondii. PLoS Pathog. *14*, e1006836.

Breckels, L.M., Mulvey, C.M., Lilley, K.S., and Gatto, L. (2016). A Bioconductor workflow for processing and analysing spatial proteomics data. F1000Res *5*, 2926.

Buchfink, B., Xie, C., and Huson, D.H. (2015). Fast and sensitive protein alignment using DIAMOND. Nat. Methods *12*, 59–60.

Campello, R.J.G.B., Moulavi, D., and Sander, J. (2013). Density-based clustering based on hierarchical density estimates. In Advances in Knowledge Discovery and Data, J.P. Mining, V.S. Tseng, L. Cao, H. Motoda, and G. Xu, eds. (Springer), pp. 160–172.

Cesbron-Delauw, M.F., Gendrin, C., Travier, L., Ruffiot, P., and Mercier, C. (2008). Apicomplexa in mammalian cells: trafficking to the parasitophorous vacuole. Traffic *9*, 657–664.

Christoforou, A., Mulvey, C.M., Breckels, L.M., Geladaki, A., Hurrell, T., Hayward, P.C., Naake, T., Gatto, L., Viner, R., Arias, A.M., and Lilley, K.S. (2016). A draft map of the mouse pluripotent stem cell spatial proteome. Nat. Commun. *7*, 8992.

Crook, O.M., Breckels, L.M., Lilley, K.S., Kirk, P.D.W., and Gatto, L. (2019). A Bioconductor workflow for the Bayesian analysis of spatial proteomics. F1000Res *8*, 446.

Crook, O.M., Mulvey, C.M., Kirk, P.D.W., Lilley, K.S., and Gatto, L. (2018). A Bayesian mixture modelling approach for spatial proteomics. PLoS Comput. Biol. *14*, e1006516.

Danne, J.C., Gornik, S.G., MacRae, J.I., McConville, M.J., and Waller, R.F. (2013). Alveolate mitochondrial metabolic evolution: dinoflagellates force reassessment of the role of parasitism as a driver of change in apicomplexans. Mol. Biol. Evol. *30*, 123–139.

Davies, H., Belda, H., Broncel, M., Ye, X., Bisson, C., Introini, V., Dorin-Semblat, D., Semblat, J.P., Tibúrcio, M., Gamain, B., et al. (2020). An exported kinase family mediates species-specific erythrocyte remodelling and virulence in human malaria. Nat. Microbiol. *5*, 848–863.

Deutsch, E.W., Csordas, A., Sun, Z., Jarnuczak, A., Perez-Riverol, Y., Ternent, T., Campbell, D.S., Bernal-Llinares, M., Okuda, S., Kawano, S., et al. (2017). The ProteomeXchange Consortium in 2017: supporting the cultural change in proteomics public data deposition. Nucleic Acids Res. *45*, D1100–D1106.

Dogga, S.K., Mukherjee, B., Jacot, D., Kockmann, T., Molino, L., Hammoudi, P.-M., Hartkoorn, R.C., Hehl, A.B., and Soldati-Favre, D. (2017). A druggable secretory protein maturase of *Toxoplasma* essential for invasion and egress. eLife *6*, e27480.

Emms, D.M., and Kelly, S. (2015). OrthoFinder: solving fundamental biases in whole genome comparisons dramatically improves orthogroup inference accuracy. Genome Biol. *16*, 157.

Engelman, D.M., Steitz, T.A., and Goldman, A. (1986). Identifying nonpolar transbilayer helices in amino acid sequences of membrane proteins. Annu. Rev. Biophys. Biophys. Chem. *15*, 321–353.

Engler, C., and Marillonnet, S. (2014). Golden Gate cloning. In DNA Cloning and Assembly Methods, S. Valla and R. Lale, eds. (Humana Press), pp. 119–131.

Frénal, K., Dubremetz, J.F., Lebrun, M., and Soldati-Favre, D. (2017). Gliding motility powers invasion and egress in Apicomplexa. Nat. Rev. Microbiol. *15*, 645–660.

Gajria, B., Bahl, A., Brestelli, J., Dommer, J., Fischer, S., Gao, X., Heiges, M., Iodice, J., Kissinger, J.C., Mackey, A.J., et al. (2008). ToxoDB: an integrated Toxoplasma gondii database resource. Nucleic Acids Res. *36*, D553–D556.

Gatto, L., Breckels, L.M., Wieczorek, S., Burger, T., and Lilley, K.S. (2014). Mass-spectrometry-based spatial proteomics data analysis using pRoloc and pRolocdata. Bioinformatics *30*, 1322–1324.

Gatto, L., and Lilley, K.S. (2012). MSnbase-an R/bioconductor package for isobaric tagged mass spectrometry data visualization, processing and quantitation. Bioinformatics *28*, 288–289.

Geladaki, A., Kočevar Britovšek, N., Breckels, L.M., Smith, T.S., Vennard, O.L., Mulvey, C.M., Crook, O.M., Gatto, L., and Lilley, K.S. (2019). Combining Lopit with differential ultracentrifugation for high-resolution spatial proteomics. Nat. Commun. *10*, 331.

Gelman, A., and Rubin, D.B. (1992). Inference from iterative simulation using multiple sequences. Statist. Sci. *7*, 457–472.

Gentleman, R.C., Carey, V.J., Bates, D.M., Bolstad, B., Dettling, M., Dudoit, S., Ellis, B., Gautier, L., Ge, Y., Gentry, J., et al. (2004). Bioconductor: open software development for computational biology and bioinformatics. Genome Biol. *5*, R80.

Gilks, W.R., Richardson, S., and Spegelhalter, D. (1995). Markov Chain Monte Carlo in Practice (Chapman & Hall/CRC).

Hakimi, M.A., Olias, P., and Sibley, L.D. (2017). Toxoplasma effectors targeting host signaling and transcription. Clin. Microbiol. Rev. *30*, 615–645.

Harding, C.R., and Meissner, M. (2014). The inner membrane complex through development of Toxoplasma gondii and Plasmodium. Cell. Microbiol. *16*, 632–641.

Havelaar, A.H., Kirk, M.D., Torgerson, P.R., Gibb, H.J., Hald, T., Lake, R.J., Praet, N., Bellinger, D.C., de Silva, N.R., Gargouri, N., et al. (2015). World Health Organization global estimates and regional comparisons of the burden of foodborne disease in 2010. PLoS Med *12*, e1001923.

Heaslip, A.T., Nishi, M., Stein, B., and Hu, K. (2011). The motility of a human parasite, Toxoplasma gondii, is regulated by a novel lysine methyltransferase. PLoS Pathog. *7*, e1002201.

Heiges, M., Wang, H., Robinson, E., Aurrecoechea, C., Gao, X., Kaluskar, N., Rhodes, P., Wang, S., He, C.Z., Su, Y., et al. (2006). CryptoDB: a Cryptosporidium bioinformatics resource update. Nucleic Acids Res. *34*, D419–D422.

Huber, W., von Heydebreck, A., Sültmann, H., Poustka, A., and Vingron, M. (2002). Variance stabilization applied to microarray data calibration and to the quantification of differential expression. Bioinformatics *18*, S96–S104.

Hunter, M.J., and Commerford, S.L. (1961). Pressure homogenization of mammalian tissues. Biochim. Biophys. Acta *47*, 580–586.

Janouškovec, J., Horák, A., Barott, K.L., Rohwer, F.L., and Keeling, P.J. (2013). Environmental distribution of coral-associated relatives of apicomplexan parasites. ISME J. *7*, 444–447.

Jung, C., Lee, C.Y.F., and Grigg, M.E. (2004). The SRS superfamily of Toxoplasma surface proteins. Int. J. Parasitol. *34*, 285–296.

Kafsack, B.F.C., Pena, J.D.O., Coppens, I., Ravindran, S., Boothroyd, J.C., and Carruthers, V.B. (2009). Rapid membrane disruption by a perforin-like protein facilitates parasite exit from host cells. Science *323*, 530–533.

Käll, L., Canterbury, J.D., Weston, J., Noble, W.S., and MacCoss, M.J. (2007). Semi-supervised learning for peptide identification from shotgun proteomics datasets. Nat. Methods *4*, 923–925.

Käll, L., Krogh, A., and Sonnhammer, E.L.L. (2004). A combined transmembrane topology and signal peptide prediction method. J. Mol. Biol. *338*, 1027–1036.

Käll, L., Storey, J.D., and Noble, W.S. (2008a). Non-parametric estimation of posterior error probabilities associated with peptides identified by tandem mass spectrometry. Bioinformatics *24*, i42–i48.

Käll, L., Storey, J.D., MacCoss, M.J., and Noble, W.S. (2008b). Assigning significance to peptides identified by tandem mass spectrometry using decoy databases. J. Proteome Res. *7*, 29–34.

Kang, S.W., Rane, N.S., Kim, S.J., Garrison, J.L., Taunton, J., and Hegde, R.S. (2006). Substrate-specific translocational attenuation during ER stress defines a pre-emptive quality control pathway. Cell *127*, 999–1013.

Karsten, V., Hegde, R.S., Sinai, A.P., Yang, M., and Joiner, K.A. (2004). Transmembrane domain modulates sorting of membrane proteins in Toxoplasma gondii. J. Biol. Chem. *279*, 26052–26057.

Katris, N.J., van Dooren, G.G., McMillan, P.J., Hanssen, E., Tilley, L., and Waller, R.F. (2014). The apical complex provides a regulated gateway for secretion of invasion factors in Toxoplasma. PLoS Pathog. *10*, e1004074.

Kats, L.M., Cooke, B.M., Coppel, R.L., and Black, C.G. (2008). Protein trafficking to apical organelles of malaria parasites - building an invasion machine. Traffic *9*, 176–186.

Koreny, L., Zeeshan, M., Barylyuk, K., Brady, D., Ke, H., Tewari, R., and Waller, R.F. (2020). Conservation of the Toxoplasma conoid proteome in Plasmodium reveals a cryptic conoid feature that differentiates between blood- and vector-stage zoite. bioRxiv https://www.biorxiv.org/content/10.1101/2020.06.26.174284v2.

Kotloff, K.L., Nataro, J.P., Blackwelder, W.C., Nasrin, D., Farag, T.H., Panchalingam, S., Wu, Y., Sow, S.O., Sur, D., Breiman, R.F., et al. (2013). Burden and aetiology of diarrhoeal disease in infants and young children in developing countries (the Global Enteric Multicenter Study, GEMS): a prospective, case-control study. Lancet *382*, 209–222.

Krogh, A., Larsson, B., von Heijne, G., and Sonnhammer, E.L.L. (2001). Predicting transmembrane protein topology with a hidden Markov model: application to complete genomes. J. Mol. Biol. *305*, 567–580.

Lacombe, A., Maclean, A.E., Ovciarikova, J., Tottey, J., Mühleip, A., Fernandes, P., and Sheiner, L. (2019). Identification of the Toxoplasma gondii mitochondrial ribosome, and characterisation of a protein essential for mitochondrial translation. Mol. Microbiol. *112*, 1235–1252.

Lebrun, M., Carruthers, V.B., and Cesbron-Delauw, M.-F. (2014). Toxoplasma secretory proteins and their roles in cell invasion and intracellular survival. In Toxoplasma Gondii, L.M. Weiss and K. Kim, eds. (Elsevier), pp. 389–453.

Lee, I., and Hong, W. (2004). RAP – a putative RNA-binding domain. Trends Biochem. Sci. *29*, 567–570.

Link, A.J., and Labaer, J. (2011). Trichloroacetic acid (TCA) precipitation of proteins. Cold Spring Harb. Protoc. *2011*, 993–994.

Long, S., Anthony, B., Drewry, L.L., and Sibley, L.D. (2017). A conserved ankyrin repeat-containing protein regulates conoid stability, motility and cell invasion in Toxoplasma gondii. Nat. Commun. *8*, 2236.

Lorenzi, H., Khan, A., Behnke, M.S., Namasivayam, S., Swapna, L.S., Hadjithomas, M., Karamycheva, S., Pinney, D., Brunk, B.P., Ajioka, J.W., et al. (2016). Local admixture of amplified and diversified secreted pathogenesis determinants shapes mosaic Toxoplasma gondii genomes. Nat. Commun. *7*, 10147.

Mulvey, C.M., Breckels, L.M., Geladaki, A., Britovšek, N.K., Nightingale, D.J.H., Christoforou, A., Elzek, M., Deery, M.J., Gatto, L., and Lilley, K.S. (2017). Using hyperLOPIT to perform high-resolution mapping of the spatial proteome. Nat. Protoc. *12*, 1110–1135.

Nielsen, H. (2017). Protein function prediction (Springer).

Parsons, H.T., Stevens, T.J., McFarlane, H.E., Vidal-Melgosa, S., Griss, J., Lawrence, N., Butler, R., Sousa, M.M.L., Salemi, M., Willats, W.G.T., et al. (2019). Separating Golgi proteins from cis to trans reveals underlying properties of cisternal localization. Plant Cell *31*, 2010–2034.

Perez-Riverol, Y., Csordas, A., Bai, J., Bernal-Llinares, M., Hewapathirana, S., Kundu, D.J., Inuganti, A., Griss, J., Mayer, G., Eisenacher, M., et al. (2019). The PRIDE database and related tools and resources in 2019: improving support for quantification data. Nucleic Acids Res. *47*, D442–D450.

Pernas, L., Adomako-Ankomah, Y., Shastri, A.J., Ewald, S.E., Treeck, M., Boyle, J.P., and Boothroyd, J.C. (2014). Toxoplasma effector MAF1 mediates recruitment of host mitochondria and impacts the host response. PLoS Biol. *12*, e1001845.

Phipson, B., and Smyth, G.K. (2010). Permutation p-values should never be zero: calculating exact p-values when permutations are randomly drawn. Stat. Appl. Genet. Mol. Biol. *9*, Article39.

R Core Team (2018). R: a language and environment for statistical computing. https://www.R-project.org/.

Rashid, M., Rashid, M.I., Akbar, H., Ahmad, L., Hassan, M.A., Ashraf, K., Saeed, K., and Gharbi, M. (2019). A systematic review on modelling approaches for economic losses studies caused by parasites and their associated diseases in cattle. Parasitology *146*, 129–141.

Roos, D.S., Donald, R.G., Morrissette, N.S., and Moulton, A.L. (1994). Molecular tools for genetic dissection of the protozoan parasite Toxoplasma gondii. Methods Cell Biol. *45*, 27–63.

Schneider, C.A., Rasband, W.S., and Eliceiri, K.W. (2012). NIH Image to ImageJ: 25 years of image analysis. Nat. Methods *9*, 671–675.

Sharpe, H.J., Stevens, T.J., and Munro, S. (2010). A comprehensive comparison of transmembrane domains reveals organelle-specific properties. Cell *142*, 158–169.

Sheiner, L., Demerly, J.L., Poulsen, N., Beatty, W.L., Lucas, O., Behnke, M.S., White, M.W., and Striepen, B. (2011). A Systematic screen to discover and analyze apicoplast proteins identifies a conserved and essential protein import factor. PLoS Pathog. *7*, e1002392.

Sheiner, L., Vaidya, A.B., and McFadden, G.I. (2013). The metabolic roles of the endosymbiotic organelles of Toxoplasma and Plasmodium spp. Curr. Opin. Microbiol. *16*, 452–458.

Sidik, S.M., Huet, D., Ganesan, S.M., Huynh, M.H., Wang, T., Nasamu, A.S., Thiru, P., Saeij, J.P.J., Carruthers, V.B., Niles, J.C., and Lourido, S. (2016). A genome-wide CRISPR screen in Toxoplasma identifies essential apicomplexan genes. Cell *166*, 1423–1435.e12.

Sievers, F., Wilm, A., Dineen, D., Gibson, T.J., Karplus, K., Li, W., Lopez, R., McWilliam, H., Remmert, M., Söding, J., et al. (2011). Fast, scalable generation of high-quality protein multiple sequence alignments using Clustal Omega. Mol. Syst. Biol. *7*, 539.

Simpson, R.J. (2010). Disruption of cultured cells by nitrogen cavitation. Cold Spring Harb. Protoc. *2010*, pdb.prot5513.

Snapp, E.L., McCaul, N., Quandte, M., Cabartova, Z., Bontjer, I., Källgren, C., Nilsson, I., Land, A., von Heijne, G., Sanders, R.W., and Braakman, I. (2017). Structure and topology around the cleavage site regulate post-translational cleavage of the HIV-1 gp160 signal peptide. eLife *6*, 1–25.

Soni, R., Sharma, D., and Bhatt, T.K. (2016). Plasmodium falciparum secretome in erythrocyte and beyond. Front. Microbiol. *7*, 194.

Striepen, B. (2013). Parasitic infections: time to tackle cryptosporidiosis. Nature *503*, 189–191.

Sun, S., Li, X., and Mariappan, M. (2020). Signal sequences encode information for protein folding in the endoplasmic reticulum. bioRxiv biorxiv.org/content/10.1101/2020.06.04.133884v1#:~:text=Summary,ER)%20via%20the%20Sec61%20translocon.&text=Signal%20sequence%20swapping%20bypasses%20BiP,chaperone%20in%20the%20ER%20lumen.

Swapna, L.S., and Parkinson, J. (2017). Genomics of apicomplexan parasites. Crit. Rev. Biochem. Mol. Biol. *52*, 254–273.

The UniProt Consortium (2019). UniProt: a worldwide hub of protein knowledge. Nucleic Acids Res. *47*, D506–D515.

Thul, P.J., Åkesson, L., Wiking, M., Mahdessian, D., Geladaki, A., Ait Blal, H., Alm, T., Asplund, A., Björk, L., Breckels, L.M., et al. (2017). A subcellular map of the human proteome. Science *356*, eaal3321.

van der Maaten, L., Hinton, G., Graham-Rowe, D., van der Maaten, L., and Hinton, G. (2008). Visualizing data using t-SNE. J. Mach. Learn. Res. *164*, 10.

Votýpka, J., Modrý, D., Oborník, M., Šlapeta, J., and Lukeš, J. (2017). Apicomplexa. In Handbook of the protists (Springer International Publishing), pp. 567–624.

Waller, R.F., and Jackson, C.J. (2009). Dinoflagellate mitochondrial genomes: stretching the rules of molecular biology. BioEssays *31*, 237–245.

Wang, Y., Lilley, K.S., and Oliver, S.G. (2014). A protocol for the subcellular fractionation of Saccharomyces cerevisiae using nitrogen cavitation and density gradient centrifugation. Yeast *31*, 127–135.

Woo, Y.H., Ansari, H., Otto, T.D., Klinger, C.M., Kolisko, M., Michálek, J., Saxena, A., Shanmugam, D., Tayyrov, A., Veluchamy, A., et al. (2015). Chromerid genomes reveal the evolutionary path from photosynthetic algae to obligate intracellular parasites. eLife *4*, e06974.

Woodcroft, B.J., McMillan, P.J., Dekiwadia, C., Tilley, L., and Ralph, S.A. (2012). Determination of protein subcellular localization in apicomplexan parasites. Trends Parasitol *28*, 546–554.

World Health Organization. (2018). World Malaria Report 2018 (World Health Organization).

Yeoh, S., O'Donnell, R.A., Koussis, K., Dluzewski, A.R., Ansell, K.H., Osborne, S.A., Hackett, F., Withers-Martinez, C., Mitchell, G.H., Bannister, L.H., et al. (2007). Subcellular discharge of a serine protease mediates release of invasive malaria parasites from host erythrocytes. Cell *131*, 1072–1083.

Young, G.A., Hinkley, D.V., and Davison, A.C. (2003). Recent developments in bootstrap methodology. Statist. Sci. *18*, 141–157.

STAR★METHODS

KEY RESOURCES TABLE

REAGENT or RESOURCE	SOURCE	IDENTIFIER
Antibodies		
Rabbit polyclonal anti-RON4	Laboratory of John Boothroyd	N/A
Rabbit polyclonal anti-MIC2	Laboratory of David Sibley	N/A
Mouse monoclonal anti-MIC3 (A80D)	Thermo Fisher Scientific	Cat#MA5-18267; RRID: AB_2539641
Rabbit polyclonal anti-BiP	Laboratory of Jay Bangs	N/A
Rabbit polyclonal anti-TOM40	Laboratory of Giel van Dooren	N/A
Rabbit polyclonal anti-CPN60	Laboratory of Boris Striepen	N/A
Rabbit polyclonal anti-GAP45	Laboratory of Dominique Soldati-Favre	N/A
Mouse monoclonal [TP3] anti-SAG1	Abcam	Cat#ab8313; RRID: AB_306466
Rabbit polyclonal anti-profilin	Laboratory of Dominique Soldati-Favre	N/A
Rabbit polyclonal anti-histone H3	Abcam	Cat#ab1791; RRID: AB_302613
Rabbit polyclonal anti-GRA1	Laboratory of Corinne Mercier	N/A
Rabbit polyclonal anti-CPL	Laboratory of Vern Carruthers	N/A
Rabbit polyclonal anti-CRT	Laboratory of Vern Carruthers	N/A
Rabbit polyclonal anti-catalase	Laboratory of Dominique Soldati-Favre	N/A
Mouse monoclonal (7E8) anti-ISP1	Laboratory of Peter Bradley	N/A
Mouse monoclonal anti-ROP1	Laboratory of John Boothroyd	N/A
Mouse monoclonal anti-V5	Invitrogen	Cat#R960-25; RRID: AB_2556564
Rat monoclonal anti-HA	Roche	Cat#ROAHAHA; RRID: AB_2687407
Peroxidase AffiniPure Goat Anti-Rabbit IgG (H+L)	Jackson Immunoresearch	Cat#111-035-003; RRID: AB_2313567
Peroxidase AffiniPure Goat Anti-Mouse IgG (H+L)	Jackson Immunoresearch	Cat#115-035-003; RRID: AB_10015289
Peroxidase AffiniPure Donkey Anti-Rat IgG (H+L)	Jackson Immunoresearch	Cat#712-035-153; RRID: AB_2340639
Goat anti-Rat IgG (H+L) secondary, Alexa Fluor 594-conjugated	Invitrogen	Cat#A-11007; RRID: AB_10561522
Goat anti-Rat IgG (H+L) secondary, Alexa Fluor 488-conjugated	Invitrogen	Cat#A-11006; RRID: AB_2534074
Goat anti-Mouse IgG (H+L) secondary, Alexa Fluor 488-conjugated	Invitrogen	Cat#A-11029; RRID: AB_2534088
Chemicals, Peptides, and Recombinant Proteins		
TMT10plex™ Isobaric Label Reagent Set, 3x0.8mg	Thermo Fisher Scientific	Cat#90111
OptiPrep™ Density Gradient Medium	Sigma Aldrich	Cat#D1556
Sequencing Grade Modified Trypsin	Promega	Cat#V5111
Benzonase Nuclease HC	Merck Millipore	Cat#71205-3
SuperSignal West Pico Chemiluminescent Substrate	Thermo Fisher Scientific	Cat#34080
cOmplete™, EDTA-free Protease Inhibitor Cocktail	Sigma Aldrich	Cat#11873580001
cOmplete™, Mini, EDTA-free Protease Inhibitor Cocktail	Sigma Aldrich	Cat#4693159001
BpiI	Thermo Fisher Scientific	Cat#ER1011
BsaI	New England Biolabs	Cat#R3535
Streptavidin, Alexa Fluor™ 594 conjugate	Invitrogen	Cta#S11227
Critical Commercial Assays		
Pierce BCA Protein Quantitation Assay	Thermo Fisher Scientific	Cat#23227

(*Continued on next page*)

Continued

REAGENT or RESOURCE	SOURCE	IDENTIFIER
Deposited Data		
Raw LC-SPS-MS^3 data, peptide and protein proteomic identification and quantification results	This paper and PRIDE Archive	PRIDE ID: PXD015269; https://doi.org/10.6019/PXD015269
hyperLOPIT spatial proteome map of *T. gondii* extracellular tachyzoite	This paper; ToxoDB.org; pRolocdata	Tables S1, S2, S3, S4, andS5; https://toxodb.org/toxo/app/record/dataset/DS_eda79f81b5; Barylyuk2020ToxoLopit
Analysed data	This paper	Tables S6, S7, S8, and S9
Experimental Models: Cell Lines		
Human: Human Foreskin Fibroblasts	Laboratory of Chris Tonkin	N/A
Toxoplasma gondii: strain RH ΔHX	Laboratory of Boris Striepen	N/A
Toxoplasma gondii: strain Δku80/TATi	Laboratory of Boris Striepen	N/A
Oligonucleotides		
Primers and oligonucleotides for the generation of transgenic *T. gondii*, see Table S10B	This paper	N/A
Recombinant DNA		
Plasmid: pGEM T-Easy	Promega	Cat#A1360
Plasmid: pPR2-HA3	Katris et al., 2014	N/A
Plasmids P1-P10 for CRISPR/Cas9-assisted and PCR-mediated genomic tagging in *T. gondii*, see Table S10A	This study	N/A
Software and Algorithms		
R	R Core Team, 2020	https://www.R-project.org/
RStudio	RStudio Team, 2020	http://www.rstudio.com/
TAGM-MAP and TAGM-MCMC	Crook et al., 2018	https://doi.org/10.18129/B9.bioc.pRoloc
t-SNE	van der Maaten et al., 2008	https://cran.r-project.org/package=Rtsne
HDBSCAN	Campello et al., 2013	https://hdbscan.readthedocs.io/en/latest/
preprocessCore	Bioconductor	https://doi.org/10.18129/B9.bioc.preprocessCore
OrthoFinder	Emms and Kelly, 2015	https://github.com/davidemms/OrthoFinder
Diamond	Buchfink et al., 2015	https://github.com/bbuchfink/diamond
Clustal Omega	Sievers et al., 2011	https://www.ebi.ac.uk/Tools/msa/clustalo/
SignalP	Almagro Armenteros et al., 2019; Nielsen, 2017	http://www.cbs.dtu.dk/services/SignalP/
TMHMM	Krogh et al., 2001	http://www.cbs.dtu.dk/services/TMHMM/
Phobius	Käll et al., 2004	http://phobius.sbc.su.se/
ImageJ	Schneider et al., 2012	https://imagej.nih.gov/ij/
softWoRx	Applied Precision	N/A
NIS-Elements	Nikon	https://www.microscope.healthcare.nikon.com/products/software/nis-elements
MassLynx	Waters	https://www.waters.com/waters/en_GB/MassLynx-MS-Software/nav.htm?locale=en_GB&cid=513662
XCalibur	Thermo Fisher Scientific	https://www.thermofisher.com/order/catalog/product/OPTON-30965#/OPTON-30965
Proteome Discoverer	Thermo Fisher Scientific	https://www.thermofisher.com/uk/en/home/industrial/mass-spectrometry/liquid-chromatography-mass-spectrometry-lc-ms/lc-ms-software/multi-omics-data-analysis/proteome-discoverer-software.html
Mascot Server	Matrix Science	http://www.matrixscience.com/server.html
Other		
Interactive interface to the annotated spatial proteome data	This paper	https://proteome.shinyapps.io/toxolopittzex/

CellPress
OPEN ACCESS

RESOURCE AVAILABILITY

Lead Contact

Further information and requests for reagents may be directed to and will be fulfilled by the Lead Contact, Ross F. Waller (rfw26@cam.ac.uk).

Materials Availability

Plasmids and *Toxoplasma* cell lines generated in this study are available upon request.

Data and Code Availability

The mass-spectrometry-based proteomics data have been deposited to the ProteomeXchange Consortium (Deutsch et al., 2017) via the PRIDE (Perez-Riverol et al., 2019) partner repository with the dataset identifier PXD015269 and https://doi.org/10.6019/PXD015269. The data are integrated into ToxoDB (https://toxodb.org/toxo/app/record/dataset/DS_eda79f81b5) (Gajria et al., 2008). The protein-level dataset generated in this study is available in the R Bioconductor pRolocdata package (version ≥ 1.25.2). An interactive interface to the annotated spatial proteome data is available through the pRolocGUI application (version 1.18.0) or via a web-based R Shiny application at https://proteome.shinyapps.io/toxolopittzex/.

EXPERIMENTAL MODEL AND SUBJECT DETAILS

T. gondii tachyzoites from the strain RH and derived strains, including RH Δku80/TATi (Sheiner et al., 2011) (a kind gift from Lilach Sheiner and Boris Striepen, The University of Georgia), were maintained at 37°C with 10% CO_2 growing in human foreskin fibroblasts (HFFs) cultured in Dulbecco's Modified Eagle Medium supplemented with 1% heat-inactivated fetal bovine serum, 10 unit ml^{-1} penicillin and 10 μg ml^{-1} streptomycin, as described elsewhere (Roos et al., 1994). When appropriate for selection, chloramphenicol was used at 20 μM and pyrimethamine at 1 μM. Scaling up of the parasite culture for hyperLOPIT experiments was done according to the method described by Roos et al. (Roos et al., 1994). Briefly, 1 ml of freshly egressed *T. gondii* culture containing approximately 5×10^6 tachyzoites was inoculated into a 25 cm^2 culture flask (T25) containing a confluent monolayer of HFFs (typically, $\sim 10^5$ cells). The parasites were allowed to propagate and egress naturally, resulting in complete lysis of the host cells in approximately 48 hours. This procedure was repeated twice to enrich the population in fast-growing parasites and increase their synchrony. The parasites emerged after the second of such passages ($\sim 5 \times 10^7$) were divided equally between two 175-cm^2 culture flasks (T175) with HFF monolayers. The cultures 'lysed out' completely after 48 hours and the harvested tachyzoites ($\sim 2 \times 5 \times 10^8$) were used to inoculate 20 T175 flasks to produce a batch of freshly egressed *T. gondii* tachyzoites ($\sim 10^{10}$ cells) for a hyperLOPIT experiment. The final yields of tachyzoites used in the three hyperLOPIT experiments are given in Table S1.

METHOD DETAILS

Generation of Transgenic *T. gondii*

We developed a CRISPR/Cas9-assisted and PCR-mediated genomic tagging strategy to perform endogenous gene tagging with epitope tags for protein localization in *T. gondii*. It involves: 1) two-step cloning of the Cas9/sgRNA construct P5 (see Table S9A in Supplemental Information for sequences and annotation of the vectors used in this study) for directing a locus-specific DNA break to facilitate homologous recombination-driven insertion of a donor DNA; 2) PCR amplification of the donor DNA fragment generating an in-frame insertion of an epitope tag as well as a drug resistance cassette amplified from one of the template vectors (P6-P10, or pPR2-HA3 (Katris et al., 2014)) using primers that include specific homology arms directing the integration of this construct into the target genetic locus (Table S9B in Supplemental Information); 3) co-transfection of the plasmid generated in step 1 and the PCR product obtained in step 2 into the parasite cells.

For tagging each gene, plasmid P5 was assembled using the Golden Gate assembly method (Engler and Marillonnet, 2014). Briefly, the sgRNA was generated by PCR amplification using a gene-specific forward primer and a general reverse primer ('Universal_sgRNA_Rv' in Table S9B) from a template Golden Gate Level M plasmid P5. The resulting sgRNA containing specific protospacer sequence (PS-sgRNA) was inserted into Golden Gate Level 1 Position 2 acceptor plasmid P4 downstream of *T. gondii* U6 promoter (obtained from P1) using BsaI sites. TgU6-PS-sgRNA cassette was then combined with the 'TgSag1 promoter – Cas9-HA-GFP – TgSag1 terminator' cassette from P2 using BpiI to create the final plasmid P5.

For C-terminal genomic tagging, we created template plasmids containing 6xHA (P6 and P9), 3xHA (P10), or 3xV5 (P7 and P8) epitope reporters and both the dihydrofolate reductase (DHFR) and the chloramphenicol acetyltransferase (CAT) resistance cassettes (P8-P10 and P6, P7, respectively) using the Golden Gate assembly method (Table S9A). For N-terminal genomic tagging with the 3xHA epitope tag, the pPR2-3HA plasmid (Katris et al., 2014) was used as a template. The resistance cassette and the reporter tag were amplified from the template plasmid using gene-specific primers that contained 3'-or 5'-end homology regions to facilitate the genomic integration by homologous recombination (Table S9B). Approximately 50 μg of plasmid P5 and 200 μl of the PCR reaction product containing the epitope tag, resistance gene, and the homology sequences were combined, ethanol-purified, and co-transfected into *T. gondii* RH Δku80/TATi as previously described (Heaslip et al., 2011). Parasites were selected with 1 μM pyrimethamine or 20 μM chloramphenicol. Individual clones were obtained by limiting dilution (Katris et al., 2014).

Immunofluorescence Microscopy and Immunoblotting

T. gondii-infected HFF monolayers grown on glass coverslips were fixed with 2% formaldehyde at room temperature for 15 min, permeabilized with 0.1% TritonX-100 for 10 min and blocked with 2% BSA for 1 h. The coverslips were then incubated with a primary antibody (see Table S9C in Supplemental Information for the list of antibodies and dilutions used) for 1 h, followed by 1 h incubation with a secondary antibody (Table S9C). Coverslips were mounted using ProLong® Diamond Antifade Mountant with DAPI (Invitrogen). Images were acquired using a Nikon Eclipse Ti widefield microscope with a Nikon objective lens (Plan APO, 100x/1.45 oil), and a Hamamatsu C11440, ORCA Flash 4.0 camera.

3D-Structured Illumination Microscopy (3D-SIM) was implemented on a DeltaVision OMX V4 Blaze (Applied Precision) with samples prepared as for widefield immunofluorescence assay (IFA) microscopy expect High Precision coverslips (Marienfeld Superior, No1.5H with a thickness of 170 μm ± 5 μm) were used in cell culture and Vectashield (Vector Laboratories) was used as a mounting reagent. Samples were excited using 405, 488, and 594 nm lasers and imaged with a 60× oil immersion lens (1.42 NA). The structured illumination images were reconstructed in softWoRx software version 6.1.3 (Applied Precision). All fluorescence images were processed using ImageJ software (Schneider et al., 2012).

For immunoblotting, performed during optimization of cell disruption and density gradient fractionation, approximately 510 × 10^7 gene-tagged parasites were purified from the host cell debris by filtration through 3-μm-pore-size polycarbonate film membrane filters (Nuclepore Track-Etch Membrane, Whatman) and collected and washed in PBS by centrifugation at 1,700 × g_{max} for 10 min at room temperature. The cell pellets were directly resuspended to an equivalent number density of approximately 5 × 10^8 ml^{-1} in NuPage LDS Sample Buffer (Thermo Fisher Scientific) supplemented with dithiothreitol (DTT) to a final concentration of 50 mM and incubated at 70°C for 10 min to extract, reduce, and denature proteins. For the hyperLOPIT density gradient assessment, aliquots of the gradient fractions containing 0.5 μg total protein were prepared in NuPAGE LDS Sample Buffer as described above. Proteins were resolved by SDS-PAGE using NuPAGE 4-12% Bis-Tris Protein Gels (Thermo Fisher Scientific) and electrotransferred onto 0.2-μm-pore-size nitrocellulose membranes (Amersham Protran Supported, GE Healthcare) using either XCell SureLock Mini-Cell with XCell II Blot Module or Mini Gel Tank with Blot Module (Thermo Fisher Scientific) according to the manufacturer's instructions. The membranes were blocked in 5% (w/v) non-fat dry milk in a tris-buffered saline solution containing 0.05% (w/v) of Tween 20 (TBST) and probed with primary and secondary antibodies (Table S9C). Protein bands were visualized via chemiluminescence detection using SuperSignal West Pico Chemiluminescent Substrate (Thermo Scientific).

Sample Preparation for hyperLOPIT

Approximately 10^{10} (Table S1 in Supplemental Information) freshly egressed extracellular tachyzoites were purified from the host cell debris by filtration through 3-μm-pore-size polycarbonate film membrane filters (Nuclepore Track-Etch Membrane, Whatman). The cells were washed with chilled PBS (pH 7.4) three times by centrifugation at 3000 × g_{max}, 4°C and resuspended to a final cell density of 5 × 10^8 ml^1 in a chilled homogenization medium (HB: 0.25 M sucrose, 10 mM HEPES•KOH pH 7.4, 1 mM EDTA) supplemented with proteinase inhibitors (cOmplete™ EDTA-free Proteinase inhibitor cocktail, Roche).

The cells were mechanically lysed by nitrogen cavitation (Hunter and Commerford, 1961; Simpson, 2010) using a Parr Instruments cell disruption vessel model 4639 (45 ml volume) at 2,000 PSI (approximately 138 bar). The system with cell suspension was allowed to equilibrate on ice for 15 min with occasional gentle agitation. The content was discharged from the vessel through the release valve at a flow rate of approximately two droplets per second. Differential centrifugation was used to return intact and poorly dispersed cell material to a subsequent cavitation cycle. The unlysed material was removed by centrifugation as described in Table S1. The resulting supernatant was considered the cell homogenate. In some cases (Table S1), the homogenate was treated with 500 U of the nuclease Benzonase (Sigma-Aldrich) for 20 min at room temperature and a further 10 min at 4°C for its viscosity to be reduced.

HyperLOPIT Subcellular Fractionation

The suspension of membrane vesicles and subcellular particles was resolved on an iodixanol density gradient as described in (Christoforou et al., 2016; Mulvey et al., 2017). Briefly, crude subcellular particles were enriched by ultracentrifugation of the homogenate underlaid with 6 and 25% (w/v) iodixanol solutions in HB for 1.5 h at 100,000 × g_{max}, 4°C (SW32Ti rotor, Optima L-80XP ultracentrifuge, Beckman) with the maximum acceleration and minimum deceleration. An aliquot of the supernatant enriched with cytosolic and soluble proteins was taken and mixed with six volumes of acetone chilled to -20°C and removed to -20°C to precipitate proteins from the solution. Opaque bands at the interfaces of the iodixanol layers containing enriched subcellular membranes and particles were collected, diluted with HB to bring the iodixanol concentration below 6% (w/v), and pelleted from residual soluble proteins by ultracentrifugation for 1 h at 200,000 × g_{max}, 4°C (SW55Ti rotor, Beckman). The pellets were resuspended in 25% (w/v) iodixanol in HB using a Dounce tissue grinder (max. volume 2 ml, Kimble, pestle A clearance 0.0030-0.0050 in., pestle B clearance 0.0005-0.0025 in.) and underlaid beneath a linear pre-formed density gradient (equal volumes of 8, 12, 16, and 18% (w/v) iodixanol solutions in HB allowed to diffuse at 4°C overnight). The sample was centrifuged for 8 h at 100,000 × g_{max}, 4°C (VTi65.1 rotor, Beckman) with the maximum acceleration and minimum deceleration allowing for isopycnic separation of subcellular particles and membranes. The resolving gradient was harvested into 23 approximately equal-volume fractions by piercing the ultracentrifugation tube bottom and allowing the liquid to dispense dropwise under gravity flow. Aliquots were taken from each fraction to determine the average density through measuring the refractive index (Eclipse Handheld Refractometer 45-02, sugar 0-32%, Billingham and Stanley), and for protein concentration assessment by the BCA protein assay (Thermo Fischer Scientific) according to the

manufacturer's instructions. The distribution of several known organelle marker proteins in the gradient fractions was assessed by Western blotting using aliquots containing 0.5 μg total protein.

Proteomic Sample Generation

In experiments Toxoplasma LOPIT 1 (TL1) and TL3, the harvested fractions of the density gradient were stored at 80°C until used; proteins were extracted from the gradient fractions by precipitation with 10% (w/v) trichloroacetic acid (TCA) as described elsewhere (Link and Labaer, 2011). In experiment TL2, each fraction of the gradient was diluted with 0.8 ml HB and centrifuged for 1 h at 100,000 × g_{max}, 4°C (TLA-55 rotor, Optima MAX-XP benchtop ultracentrifuge, Beckman). The supernatant was carefully aspirated and discarded, membrane pellets were resuspended in 0.8 ml HB by repeated tube inversion and pelleted again by ultracentrifugation. The supernatant was discarded, and the resulting membrane-enriched pellets were stored at -80°C until used. Protein (TL1 and TL3) or membrane (TL2) pellets, including the acetone-precipitated proteins from the cytosol-enriched fraction, were resolubilized in triethylammonium bicarbonate (TEAB) buffered solution (pH 8.3) containing either 0.1% SDS (TL1) or 8 M urea, 0.2% SDS (TL2 and 3) assisted by sonication (5 cycles of 30 s ON, 30 s OFF at high power, Bioruptor Plus ultrasonic disintegrator, Diagenode). Protein concentration was measured by the BCA assay.

Sequential gradient fractions were aggregated to nine pools containing 60 to 100 μg protein and maximizing distinct subcellular marker protein distributions based on Western blots analysis (see pooling strategies in Table S1). A tenth fraction was derived from the soluble protein-containing fraction. Proteins were reduced with 10 mM Tris(2-carboxyethyl)phosphine (TCEP; Sigma-Aldrich) for 1 h at room temperature followed by alkylation of cysteine residue side chain thiol groups with iodoacetamide (Sigma-Aldrich) at approximately 17 mM final concentration for 30 min at room temperature in the dark. Six volumes of pre-chilled (-20°C) acetone were added to the reaction mixtures and proteins were allowed to precipitate overnight at -20°C. The samples were centrifuged at 16,000 × g_{max} for 10 min at 4°C, the supernatant was carefully aspirated and discarded, and the protein pellets were air-dried at room temperature for 5 min.

Acetone-precipitated protein pellets were resuspended in 100 mM TEAB-buffered solution (pH 8.3) with the assistance of sonication (Bioruptor Plus, Diagenode, 5 cycles of 30 s ON, 30 s OFF, high power) and digested with 1 μg of sequencing-grade trypsin (Promega) for 2 h at 37°C followed by the addition of another 1 μg aliquot of the enzyme and incubation at 37°C overnight. The digests were centrifuged for 10 min at 16,000 × g_{max} at 4°C to remove any insoluble material, and the supernatants were transferred to a new 1.5 ml Protein LoBind microcentrifuge tube (Eppendorf) and labeled with TMT10plex isobaric tagging reagents (Thermo Fisher Scientific) according to the manufacturer's instructions. Briefly, 0.8 mg of TMT10plex reagents were brought to room temperature and dissolved in 41 μl of LCMS-grade acetonitrile immediately before use. The peptide digest samples (approximately 100 μl) were transferred to the TMT10plex reagent vials and the reaction mixtures were incubated at room temperature for 1-2 h with constant agitation (800 RPM, PHMT thermomixer, Grant Bio Instruments). The reaction was stopped by adding 8 μl of 5% (v/v) hydroxylamine solution and incubation for 15 min at room temperature with agitation. The TMT-labelled fractions were combined and reduced to dryness in a refrigerated (4°C) vacuum centrifuge (Labconco).

The combined TMT-labelled peptide samples were desalted using C18 solid-phase extraction (SPE) cartridges (SepPak C18, 100 mg sorbent, Waters). The dry samples were resuspended in 0.8 ml of 0.5% (v/v) trifluoroacetic acid (TFA) solution in HPLC-grade water with the assistance of sonication (Bioruptor Plus, Diagenode, 5 cycles of 30 s ON, 30 s OFF, high power). The SPE resin was conditioned with 1.6 ml of LCMS-grade acetonitrile and equilibrated in 0.1% (v/v) aqueous TFA solution (a total volume of 1.6 ml). The peptide samples were loaded onto the cartridges under the gravity-flow. The cartridges were washed with 1.6 ml of 0.1% (v/v) aqueous TFA solution to remove salts and other polar low-molecular-weight contaminants and equilibrated in 0.5% (v/v) aqueous solution of acetic acid (a total volume of 1.6 ml). The peptides were eluted from the resin using 1.6 ml of 70% (v/v) LCMS-grade acetonitrile, 0.5% (v/v) acetic acid solution in HPLC-grade water, and reduced to dryness in a refrigerated (4°C) vacuum centrifuge (Labconco).

Chromatographic Prefractionation of Peptides

The TMT10plex-labelled desalted peptide samples were fractionated by high-pH reverse-phase chromatography on an Acquity UPLC BEH C18 column (2.1-mm i.d. × 150-mm; 1.7-μm particle size) with a VanGuard pre-column (2.1 × 5 mm) packed with the same resin (both from Waters) using an Acquity UPLC system equipped with an autosampler, a binary solvent manager, and a diode array detector (Waters). The following solutions for gradient elution were used: 20 mM ammonium formate in HPLC-grade water, pH 10 (Eluent A); 20 mM ammonium formate in LCMS-grade acetonitrile : HPLC-grade water 80:20 (v/v), pH 10 (Eluent B).

The dried peptide samples were resuspended in 100 μL of 5% (v/v) Eluent B in Eluent A, sonicated (5 cycles of 30 s ON, 30 s OFF, high power, Bioruptor Plus, Diagenode), spun for 10 min at 16,000 × g_{max} to remove any insoluble material, and the supernatants were injected onto the column equilibrated with at least 20 column volumes of 95% Eluent A : 5% Eluent B. A flow rate of 0.244 ml min^{-1} was maintained. The percentage of Eluent B was varied according to the following program: 5% for 10 min, 5 to 75% over 50 min, a ramp to 100% over 2 min followed by 5.5 min at 100%, switching to 5%, and equilibration for 10 min. Fifty 1-min fractions were collected along the elution profile of the peptides (approximately from minute 10 to 60 of the program) and reduced to dryness. For the downstream LC-MS analysis, the fractions corresponding to each TMT10plex set were concatenated into 15-18 samples by combining pairs of fractions which eluted at different time points during the gradient, e.g., fraction 1, 16, and 31, fraction 2, 17, and 32, etc.

LC-MS Analysis of Peptides

All mass spectrometry analyses were performed on an Orbitrap Fusion™ Lumos™ Tribrid™ instrument coupled to a Dionex Ultimate™ 3000 RSLCnano system (Thermo Fisher Scientific) as described in (Geladaki et al., 2019).

Briefly, each of the fractionated samples was resuspended in 30 μL of 0.1% (v/v) aqueous solution of formic acid. Approximately 1 μg of peptides was loaded per injection for LC-MS/MS analysis.

The nano-flow liquid chromatography method for LC-MS/MS was set as follows. Eluent A was 0.1% (v/v) formic acid solution in water. Eluent B was 80% (v/v) aqueous acetonitrile supplemented with formic acid to a final concentration of 0.1% (v/v). The sample loading solvent was 0.1% (v/v) formic acid in water. All solvents and reagents were of HPLC gradient grade or better. Peptides were loaded onto a micro precolumn (300 μm i.d. × 5 mm, particles were C18 PepMap 100, 5 -μm particle size, 100 Å pore size, Thermo Fisher Scientific) using the loading pump for 3 min. After this, the valve was switched from load to inject. Peptides were separated on a Proxeon EASY-Spray column (PepMap RSLC C18, 50 cm × 75 μm i.d., 2 μm particle size, 100 Å pore size, Thermo Fisher Scientific) using a 2-40% (v/v) gradient of acetonitrile supplemented with 0.1% (v/v) formic acid at 300 nL min^{-1} over 93 min. A wash step (90% Eluent B for 5 min) was included, followed by re-equilibration into Eluent A. The total run time was 120 min.

The MS workflow parameters were set as follows using the Method Editor in XCalibur v3.0.63 (Thermo Fisher Scientific) for the SPS-MS^3 acquisition method. Detector type: Orbitrap; Resolution: 120,000; Mass range: Normal; Use quadrupole isolation: Yes; Scan range: 380-1,500; RF lens: 30%; AGC target: 4e5; Max inject time: 50 ms; Microscans: 1; Data type: Profile; Polarity: Positive; Monoisotopic peak determination: Peptide; Relax restrictions when too few precursors are found: Yes; Include charge state(s): 2-7; Exclude after n times: 1; Exclusion duration (s): 70; Mass tolerance (p.p.m.): Low: 10; high: 10; Exclude isotopes: Yes; Perform dependent scan on single charge state per precursor only: Yes; Intensity threshold: 5.0e3; Data-dependent mode: Top speed; Number of scan event types: 1; Scan event type 1: No condition; MSn level: 2; Isolation mode: Quadrupole; Isolation window (*m/z*): 0.7; Activation type: CID; CID collision energy (%): 35; Activation Q: 0.25; Detector type: Ion trap; Scan range mode: Auto; *m/z*: Normal; Ion trap scan rate: Turbo; AGC target: 1.0e4; Max inject time (ms): 50; Microscans: 1; Data type: Centroid; Mass range: 400-1200; Exclusion mass width: *m/z*: Low: 18; high: 5; Reagent: TMT; Precursor priority: Most intense; Scan event type 1: No condition; Synchronous precursor selection: Yes; Number of precursors: 10; MS isolation window: 0.7; Activation type: HCD; HCD collision energy (%): 65; Detector type: Orbitrap; Scan range mode: Define *m/z* range; Orbitrap resolution: 60,000; Scan range (*m/z*): 100-500; AGC target: 1.0e5; Max inject time (ms): 120; Microscans: 1; Data type: Profile; AGC, automatic gain control; HCD, higher-energy collisional dissociation; CID, collision-induced dissociation.

An electrospray voltage of 2.1 kV was applied to the eluent via the electrode of the EASY-Spray column. The mass spectrometer was operated in positive ion data-dependent mode for SPS-MS^3. The total run time was 120 min.

QUANTIFICATION AND STATISTICAL ANALYSIS

Raw LC-MS Data Processing and Quantification

Raw LC-MS data files were processed with Proteome Discoverer v2.1 (Thermo Fisher Scientific) using the Mascot server v2.6.0 (Matrix Science). The annotated protein sequences for *T. gondii* strain ME49 (reference strain) retrieved from the ToxoDB.org database (release 29, downloaded on 12.10.2016) was used along with common contaminants from the common Repository of Adventitious Proteins (cRAP) v1.0 (112 sequences, adapted from the Global Proteome Machine repository, https://www.thegpm.org/crap/). Precursor and fragment mass tolerances were set to 10 ppm and 0.8 Da, respectively. Trypsin was set as the enzyme of choice and a maximum of 2 missed cleavages were allowed. Static modifications were carbamidomethyl (C), TMT6plex (N-term), and TMT6plex (K). Dynamic modifications were oxidation (M), deamidated (NQ), TMT6plex (S/T). Percolator version 2.05 (Käll et al., 2007, 2008b, 2008a) was used to assess the false discovery rate (FDR) and only high-confidence peptides were retained.

Quantification at the MS^3 level was performed within the Proteome Discoverer workflow using the Most Confident Centroid method for peak integration and integration tolerance of 20 p.p.m. An isolation interference threshold was set to 50%. Reporter ion intensities were adjusted to correct for the isotopic impurities of the different TMT reagents (manufacturer's specifications).

Protein grouping was carried out according to the strict parsimony principle. Only proteins with a full reporter ion series and medium ($q \leq 0.05$) or high ($q \leq 0.01$) FDR confidence level were retained. Non-*Toxoplasma* proteins were removed for downstream analysis.

Location Prediction by Machine Learning Methods

Data analysis was performed using the R (R Core Team, 2018) Bioconductor (Gentleman et al., 2004) packages *MSnbase* v2.8.3 (Gatto and Lilley, 2012) and *pRoloc* v1.22.1 (Gatto et al., 2014) as described in (Breckels et al., 2016; Crook et al., 2019). Briefly, the quantitative proteomics datasets obtained in three independent hyperLOPIT experiments were subset for shared proteins with full TMT10plex quantitation data series, thereby yielding a concatenated dataset of 3,832 features (proteins) quantified across 30 samples (TMT10plex intensity values). The raw quantitation values output from Proteome Discoverer were normalized feature-wise to the sums of intensities across samples followed by variance-stabilizing normalization (Huber et al., 2002). Principle component analysis (PCA) and t-distributed Stochastic Neighbour Embedding (t-SNE) (van der Maaten et al., 2008) were used for dimensionality reduction and data visualization.

For t-SNE, the data were preprocessed as follows. First, the normalized data were centered, scaled, and PCA-transformed. The top principal components accounting for a cumulative variance of approximately 99% were filtered and subjected to a statistical

whitening transformation such that each principal component had variance 1. An embedding of the preprocessed data into two dimensions was produced using a perplexity of 50 and exact gradient calculation for a maximum of 10,000 iterations. The computed coordinates were recorded and used to obtain the two-dimensional data projection shown throughout the text.

Unsupervised clustering of proteins was performed on the normalized abundance data using the HDBSCAN* algorithm (Campello et al., 2013) available through the Python library *hdbscan* (https://hdbscan.readthedocs.io/en/latest/index.html). We used the distance to the 8th nearest neighbor to define the core distance for each protein (min_samples = 8) and allowed a minimum of 13 proteins per cluster (min_cluster_size = 13). Flat clusters from the cluster tree hierarchy were selected using 'leaf'. All other parameters were kept at default values. The result was verified to include no clusters with zero stability ($\lambda > 0$).

For the Bayesian machine-learning classification of protein locations, a set of 718 manually curated marker proteins defining 26 subcellular classes was compiled using previously published data or information available from public sources such as gene annotation in ToxoDB.org, ApiLoc database (http://apiloc.biochem.unimelb.edu.au/apiloc/apiloc), Library of Apicomplexan Metabolic Pathways (http://www.llamp.net/), and KEGG pathway database (https://www.genome.jp/kegg/) (656 proteins), and in-house protein localization by epitope-tagging and immunofluorescence microscopy (62 proteins). A Bayesian generative classifier based on t-augmented Gaussian mixture models (TAGM) was used to probabilistically attribute proteins marked as '*unknown*' to the 26 classes defined by the marker set using maximum *a posteriori* prediction (TAGM-MAP) or Markov-chain Monte-Carlo (TAGM-MCMC) methods as described in (Crook et al., 2019). TAGM-MAP was used to obtain the maximum *a posteriori* probability of each protein to belong to one of the 26 classes or an outlier component. We determined the model parameters by performing 100 iterations of the expectation-maximization (EM) algorithm using the default priors and confirmed convergence by assessing the log-posterior plot. The class with the highest TAGM-MAP allocation probability was defined as the most likely protein subcellular location. To retain only high-confidence assignments, a threshold of 99% was set on the posterior localization probability, which was defined as a product of the allocation probability and the complement of the outlier probability: $p_{localisation} = p_{allocation} \cdot (1 - p_{outlier})$. To quantify uncertainty in the allocation of proteins to organelles, we applied TAGM-MCMC. To obtain samples from the posterior localization distributions, we performed inference in this model using Markov-chain Monte-Carlo (Gilks et al., 1995). The collapsed Gibbs sampler was run in parallel for 9 chains, with each chain run for 25,000 iterations. We discarded 10,000 iterations for burn-in and thinned the chain by retaining every 20th sample. For the combined 3 replicate experiment, we discarded 5 chains because they were deemed not to have converged from visual inspection. We used the Gelman-Rubin diagnostic (Gelman and Rubin, 1992) to further assess the convergence of the remaining Markov chains. We computed a potential scale reduction factor ($\widehat{R}$) of 1.02, which is less than 1.2, upon which we concluded the convergence of our algorithm. The 750 samples from each of these 4 chains are then pooled together for further downstream processing.

Prediction of Signal Peptides and TM Spans

SignalP 5.0 (Almagro Armenteros et al., 2019) was used to predict cleaved signal peptides in *T. gondii* ME49 annotated protein sequences with the organism group set to Eukarya.

Transmembrane (TM) span prediction was performed using TMHMM 2.0 (Krogh et al., 2001). It is known that signal peptides can create TM false positives, so the results of TMHMM and SignalP analyses were compared and whenever a protein was predicted to have an N-terminal TM span and simultaneously a signal peptide the first TM span predicted by TMHMM was removed (Table S3).

Distribution of Gene and Protein Characteristics

The TAGM-MAP-predicted subcellular proteomes (localization probability > 99%) were used to quantitatively assess the distributions of the calculated protein pI (Table S6A), the protein-averaged ratio of non-synonymous to synonymous mutation rates (d_N/d_S) (Lorenzi et al., 2016) (Table S6B), the density of single nucleotide polymorphism (SNP density; data retrieved from the ToxoDB.org; Table S6C), and the average phenotype score determined by Sidik et al. in the whole-genome CRISPR/Cas9-mediated loss-of-fitness screen in *T. gondii* (Sidik et al., 2016) (Table S6D). These analyses sought to identify subcellular proteomes whose distributions of these quantities are unusual given the spatial proteome map described here. No prior knowledge of the true distributions of these quantities was available, hence, a non-parametric significance test based on an exact inference of null distributions of class-specific means through random permutation of class labels was performed.

In brief, for each of the analyzed quantities, the vector of organelle class labels was randomly permuted with replacement, and the mean value was computed for each subcellular class. The procedure was repeated $m = 10^6$ times to infer the null distributions of class means. To test the hypothesis that the observed distribution of the quantity could emerge by chance, the number b of instances from the null distribution at least as extreme as the observed mean was counted. The approximate *p-value* was then calculated according to the following formula: $p = (b+1)/(m+1)$ (Phipson and Smyth, 2010; Young et al., 2003). The resulting *p-value*s were adjusted for multiple comparisons using the Benjamini-Hochberg method (Benjamini and Hochberg, 1995).

Analysis of Gene Co-expression

RNA-Seq gene expression data sets were collected from ToxoDB (Table S5). All data were downloaded as FPKM values. In parallel, the combined data sets were treated in three different ways: i) non-normalized, ii) z-transformed, and iii) quantile normalized. Quantile normalization was done using the 'normalize.quantiles' function in the R package preprocessCore (https://github.com/bmbolstad/preprocessCore). Co-expression levels of genes across the data sets were calculated as both Pearson and Spearman rank correlations.

To test if genes within a given cluster showed signs of co-expression, all pair-wise co-expression values between members of the cluster were compared to the co-expression between members of the cluster and all genes outside the cluster. The distributions of co-expression levels were compared using the Mann-Whitney *U* test of medians, and Cohen's *d* test of effect size. All tests were carried out in R (R Core Team, 2018). All normalization procedures and correlation measures yielded highly similar results (Table S5).

Similarly, to test gene co-expression between subcompartments of subcompartment-resolved organelles, all pair-wise co-expression values between members of the subcompartment clusters (e.g., one gene selected from *apical 1* and one from *apical 2*) were compared to the co-expression values between members of the organelle (i.e., *apical 1* or *apical 2*) and all genes outside the organelle (not *apical 1* or *apical 2*). This analysis was performed for the apical complex, ER, mitochondrion, nucleus, plasma membrane, and rhoptries using the same statistical tests and data normalization as above (Table S5).

Gene Orthology Analysis

Eukaryote-wide protein orthogroups were defined using OrthoFinder (Emms and Kelly, 2015) between 79 proteomes spanning 12 levels of evolutionary divergence (Table S8A) using default OrthoFinder parameters. Either predicted or reviewed proteomes were downloaded from protein databases UniProt (The UniProt Consortium, 2019), ToxoDB (Gajria et al., 2008), PlasmoDB (Aurrecoechea et al., 2009), PiroplasmaDB (Aurrecoechea et al., 2010), and CryptoDB (Heiges etal., 2006). Proteomes were compared in all vs. all searches using Diamond (Buchfink et al., 2015), and *T. gondii* protein orthologues were inferred under stringent criteria (reciprocal best hits, RBHs) in all 78 species (Emms and Kelly, 2015). A binary bit string representing the absence/presence profile of each *T. gondii* protein orthologue at all evolutionary levels was computed from the OrthoFinder output (Tables S8B and S8C). A conservation score for each *T. gondii* protein against a predefined set binary conservation profiles (Table S8D) was computed using the pairwise Jaccard index. Based on the highest conservation score, each *T. gondii* protein was assigned a conservation profile (Table S8E).

To test for evidence of cell compartments enriched in new orthogroups at a given phylogenetic position, protein sets for each conservation profile were then tested for enrichment across all of the hyperLOPIT-derived annotation classes and a *p-value* for the likelihood of a given enrichment to have occurred by chance was obtained using a hypergeometric test (Table S8F).

Properties of Signal Peptides and TM Spans

Analyses of the signal peptide (SP) and transmembrane (TM) span sequence properties were performed as previously described (Parsons et al., 2019) with only minor modifications. SP-containing proteins and monotopic integral membrane proteins were identified using SignalP 4.1 (Nielsen, 2017) and TMHMM 2.0 as described above. Phobius (Käll et al., 2004) was used to estimate the initial TM span edge positions and the cytoplasm-exoplasm transmembrane topology.

T. gondii protein sequences were augmented with sequence information from close apicomplexan homologs identified in our OrthoFinder-based search for gene homologs across 79 eukaryotic taxa (see above). Resulting family groups all had a single, consistent organelle or subcompartment annotation that was derived from the *T. gondii* query protein.

Families of sequences were multiply aligned using Clustal Omega (Sievers et al., 2011) with default parameters. TM span edge positions were refined using the multiple alignment of each homolog family. First, the edges of the TM span (initially predicted by Phobius) were adjusted within a region of five residues either side by selecting the point in the alignment with the maximum difference in GES-scale (Engelman et al., 1986) hydrophobicity (summed over all proteins in the alignment) between the adjacent five residues on the side of the TM span and the adjacent five residues on the opposite side. Next, the edge positions were trimmed or extended according to the average hydrophobicity over the whole alignment. If the mean hydrophobicity of the next residue exceeded 1.0 kcal mol^{-1} (Gly or more hydrophobic), the edge was extended. Similarly, if the mean hydrophobicity of an edge residue was below 1.0 kcal mol^{-1}, the edge was trimmed. Finally, individual protein adjustments were made, extending or trimming positions for each span sequence. Accordingly, individual TM span edges were trimmed if they ended in a gap or a hydrophilic residue (defined here as Arg, Lys, Asp, Glu, Gln, Asn, His, or Ser) or extended if the next residue was suitably hydrophobic (Phe, Met, Ile, Leu, Val, Cys, Trp, Ala, Thr, or Gly).

Next, families of proteins were multiply aligned again using Clustal Omega, and the following additional checks were made for a comparable TM span, comparing each OrthoFinder hit with the query: (1) the length of the protein must not differ by more than 200 residues; (2) there must not be more than four gap insertions in the TM span region; (3) the separation from the TM span to the N-terminus must not differ by more than 75 residues; and (4) there must be a cursory similarity between span sequences (mean, aligned regional BLOSUM62 score > 0.8).

SP cleavage site positions were taken from the SignalP predictions.

The resulting sequence counts across compartment-specific protein families are given in Table S7A; Table S7C.

Given that families contain different numbers of protein sequences with different degrees of similarity, each protein was weighted according to its dissimilarity to all other sequences in the whole data set. Dissimilarity weights for each protein (w_p) were obtained using a BLAST+ search of each sequence (maximum e-value 10^{-20}) against a database of all the protein sequences and were calculated as:

$$w_p = \frac{1}{\sum_{i=1}^{N_p} \frac{s_i}{m_i^p}}$$

Here, s_i is the BLAST+ bit score of the aligned high-scoring database hit i (from a total of N_p hits) and m_i^p is the maximum possible bit score value, the bit score if the query were compared with itself over the same alignment region. Accordingly, the dissimilarity weight is 1.0 if the search only finds itself and approximately $1/N$ if it finds N very similar sequences. This protects against large and/or well-conserved protein families having an undue influence on the measurement of general TM span or SP properties.

The distributions of TM span length of compartment-specific protein families were compared pairwise using the Mann-Whitney *U* test. The resulting *p-value*s (smaller means less similarity between the distributions) were used to cluster the distributions by Ward's method (Figure 5B).

The positional abundance of amino acid residue types in SP sequences (Table S7B) was analyzed for the region of 15 residues [-10, +5] anchored on the signal cleavage site using Fisher's exact test on the counts of each residue type (vs. other types) in a query compartment-specific protein set compared to all other protein sets. All counts have been corrected for familial similarity using the measure of uniqueness described above and re-scaling for the original protein count.

Protein Sequence Logo Plots

The frequency of residue occurrence in SPs and flanking regions of *T. gondii* and their close homologs was visualized using logo plots. Logo plots were generated by specially written Python scripts (available at github.com/tjs23/logo_plot), after randomly sampling 1000 sequences for each data set, from position-specific residue abundance probabilities calculated from dissimilarity-weighted sequences. Sampling sequences using dissimilarity weights (as defined above) reduced the effect of similar, redundant sequences and allowed better comparison of groups containing differently sized homologous protein families. Different proteins within each subgroup were aligned by anchoring their sequences at the SP cleavage site, before the generation of logo plots (Figures 5A and S6).

Cell Host & Microbe, Volume *28*

Supplemental Information

A Comprehensive Subcellular Atlas of the *Toxoplasma* Proteome via hyperLOPIT Provides Spatial Context for Protein Functions

Konstantin Barylyuk, Ludek Koreny, Huiling Ke, Simon Butterworth, Oliver M. Crook, Imen Lassadi, Vipul Gupta, Eelco Tromer, Tobias Mourier, Tim J. Stevens, Lisa M. Breckels, Arnab Pain, Kathryn S. Lilley, and Ross F. Waller

A comprehensive subcellular atlas of the *Toxoplasma* proteome via hyperLOPIT provides spatial context for protein functions

Konstantin Barylyuk, Ludek Koreny, Huiling Ke, Simon Butterworth, Oliver M. Crook, Imen Lassadi, Vipul Gupta, Eelco Tromer, Tobias Mourier, Tim J. Stevens, Lisa M. Breckels, Arnab Pain, Kathryn S. Lilley, and Ross F. Waller

Supplemental Information

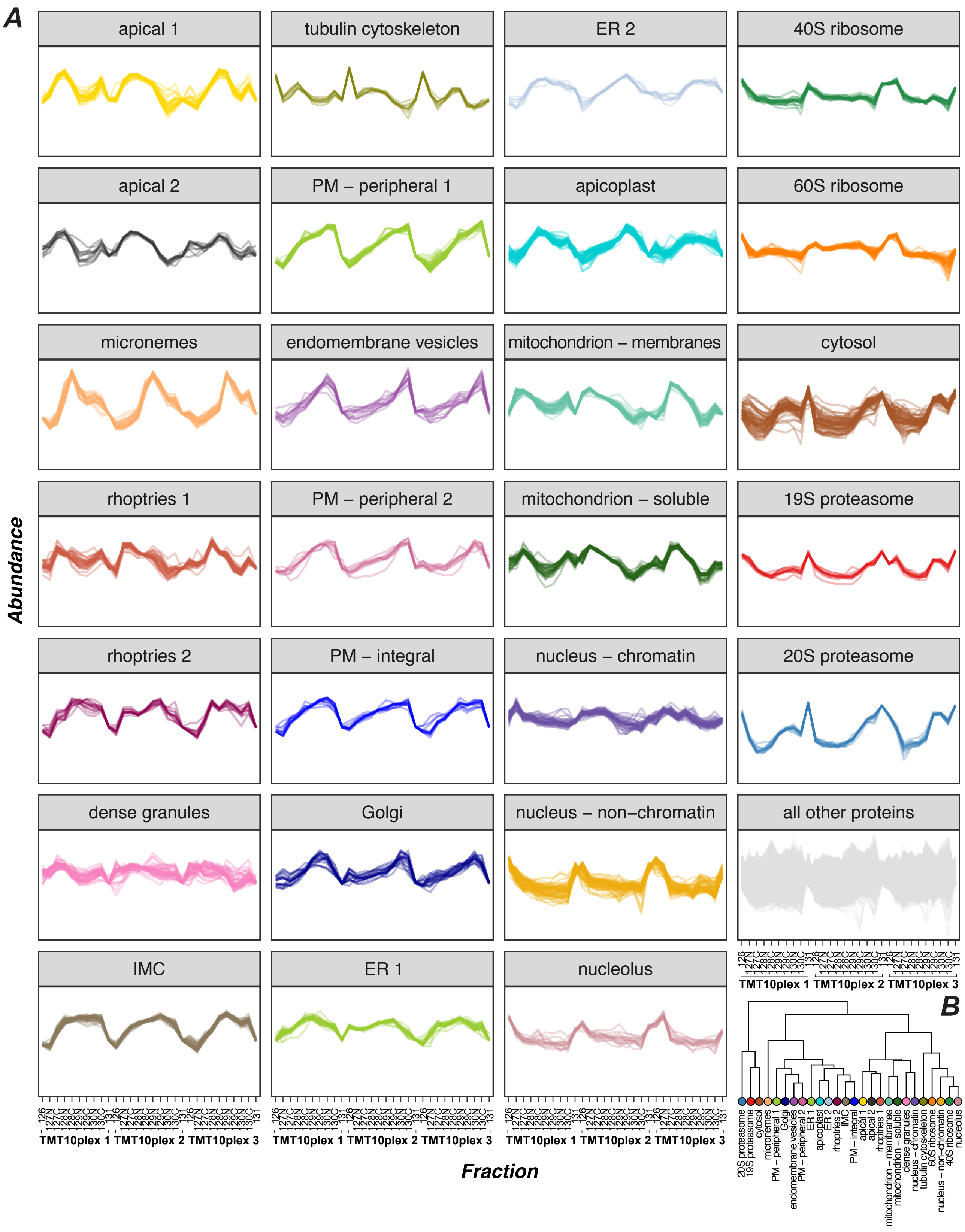

Figure S1. Abundance distribution profiles of marker and unknown proteins measured across three hyperLOPIT experiments, related to Figure 1.

A. The normalized intensities are shown on the Y-axis (Abundance). Data from three 10plex hyperLOPIT experiments are concatenated to yield a single 30plex dataset. The fractions are labelled according to the TMT10plex tag used for labelling.

B. A dendrogram of hierarchical clustering of marker protein abundance distribution profiles. For each subcellular class, a consensus abundance distribution profile was generated by averaging the profiles of the respective marker proteins. Hierarchical clustering was performed using Euclidean distance and unweighted pair group method with arithmetic mean (UPGMA)

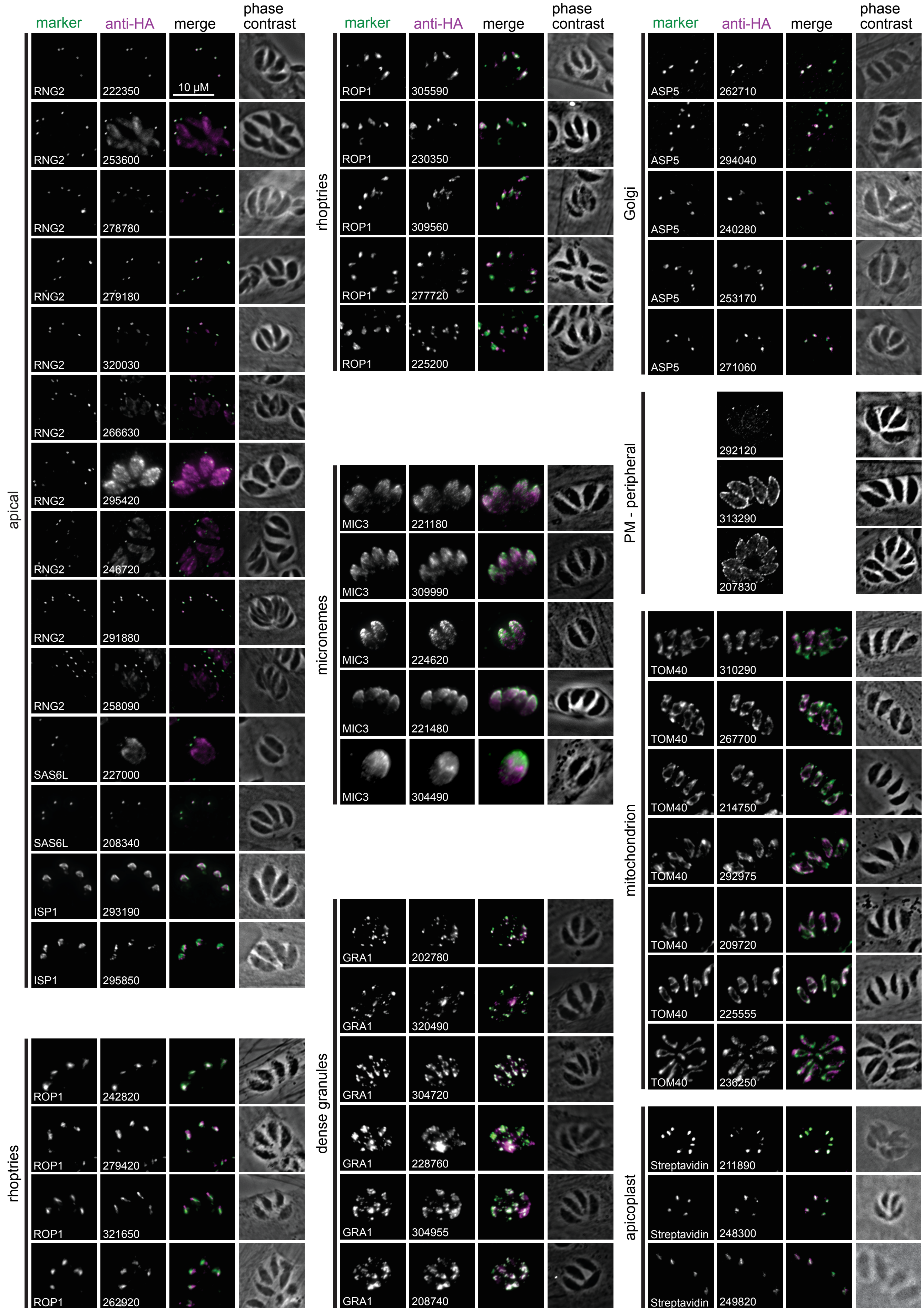

Figure S2. Validation of hyperLOPIT-predicted subcellular locations of select uncharacterized proteins by epitope tagging and immunofluorescence microscopy, continued from Figure 2.

Scale bar = 10 µm for all.

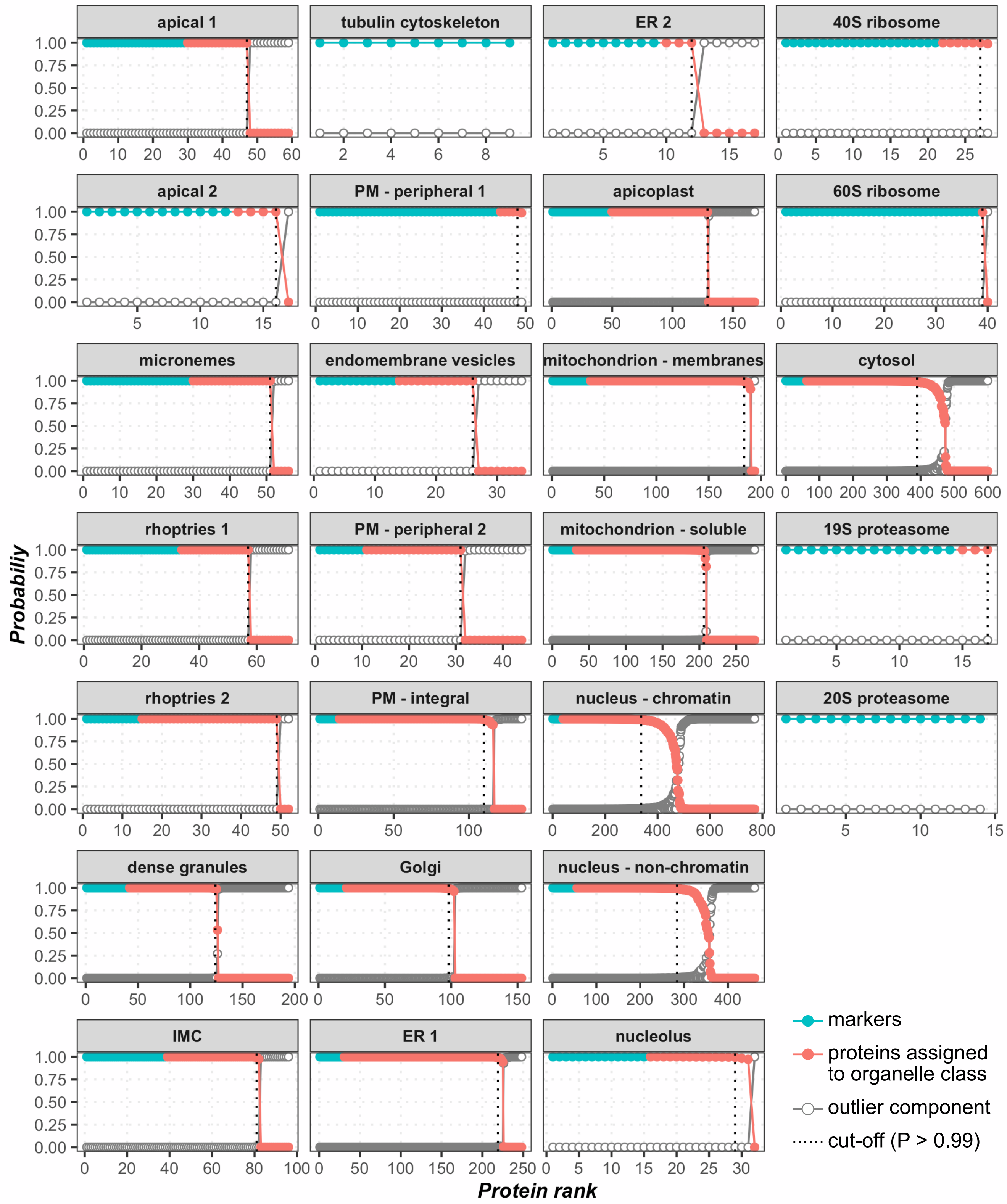

Figure S3. The posterior localization probabilities of 3,832 *T. gondii* proteins determined by a supervised Bayesian classification method TAGM-MAP, related to Figure 3.

Proteins are grouped by the most probable subcellular class, as per the TAGM-MAP classification result, and ranked on the x-axis by their localization probability. The marker proteins are shown in cyan, and the allocated proteins are in red. For each protein, the probability to belong to the outlier component is also shown in grey. The vertical dotted line in each panel indicates the protein localization prediction cutoff (localization probability threshold of 0.99). Only proteins with the localization probability above the threshold of 0.99, i.e. with the rank below the cutoff, retained their class label, whereas the rest of the proteins were labelled as ‘*unassigned*’.

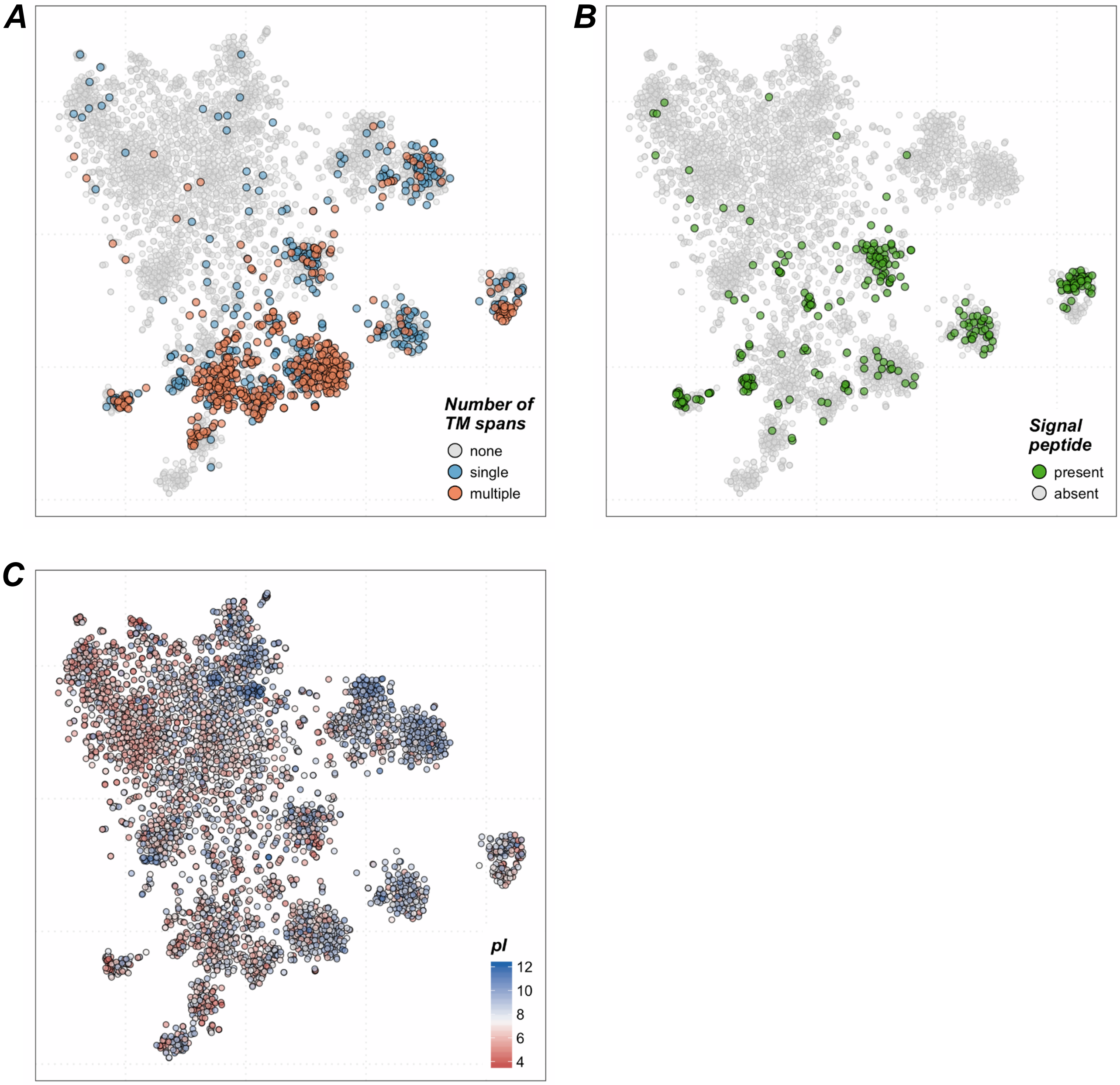

Figure S4. Distributions of select protein sequence features and properties in the spatial proteome of *T. gondii* extracellular tachyzoite, related to Figures 3, and 5.

A. A t-SNE projection of the 30plex hyperLOPIT data on 3,832 *T. gondii* proteins with monotopic (blue) and polytopic (red) integral membrane proteins highlighted. TMHMM 2.0 was used to predict transmembrane (TM) spans. The TM spans that overlapped with the signal peptide predicted by SignalP were removed.

B. Same as in ***A*** but with the proteins predicted to have a signal peptide (SignalP 5.0) highlighted in green.

C. Same as in ***A*** but showing the distribution of protein charge. Proteins are colored according to protein pI computed based on the amino acid sequence. The scale is from red for acidic proteins to blue for basic proteins with the midpoint at pI = 7.4 (colorbar in the bottom-right corner of the pane

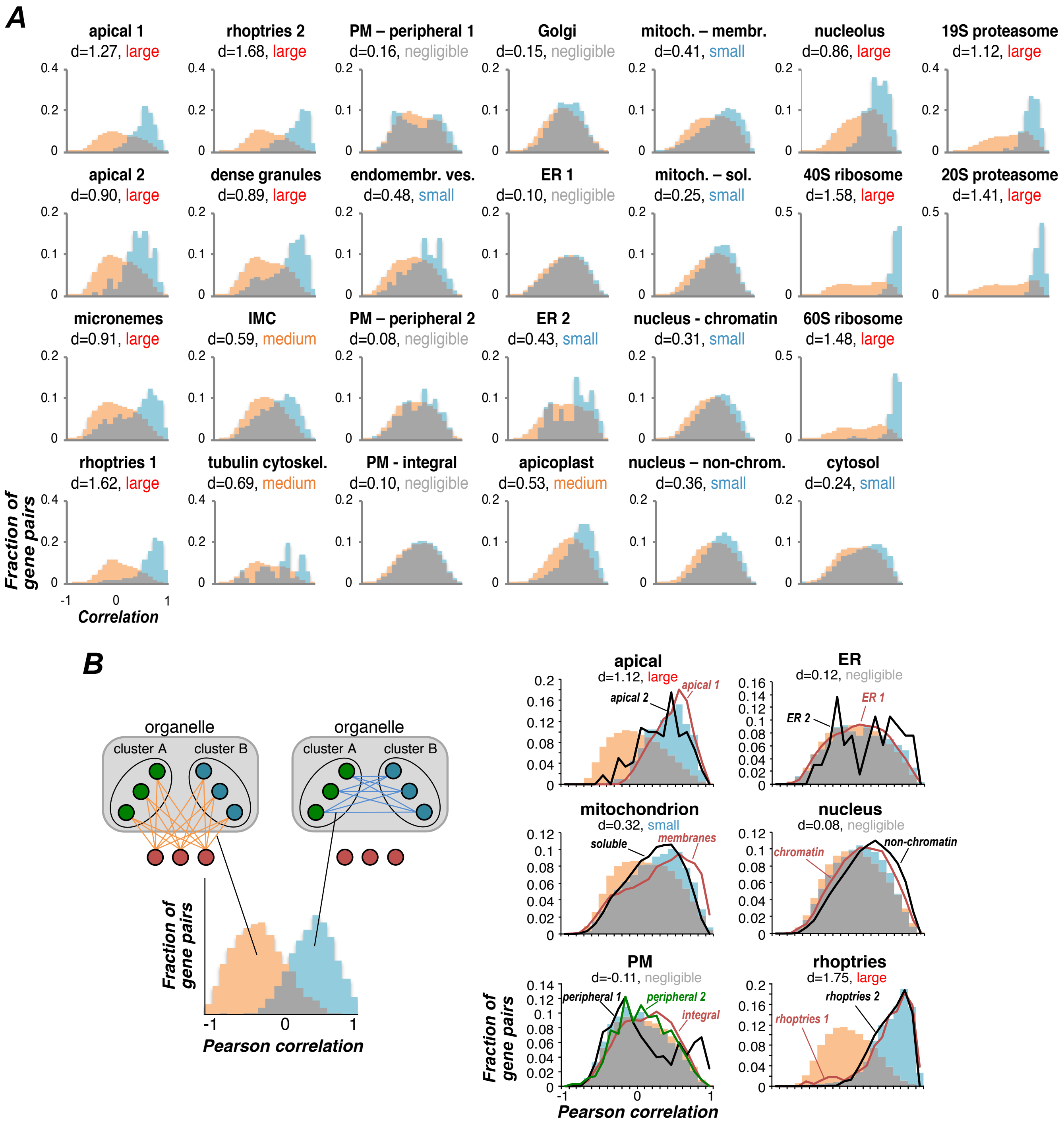

Figure S5. Gene co-expression patterns across *T. gondii* subcellular landscape, related to Figure 4.

A. Gene co-expression levels for 26 gene clusters corresponding to *T. gondii* extracellular tachyzoite subcellular compartments determined by hyperLOPIT. The co-expression levels for the genes within hyperLOPIT-defined cluster are shown as light-blue bars in histograms. The co-expression levels between the cluster members and all the genes that are not members of the cluster are shown as orange bars.

B. Gene co-expression levels within subcompartment gene clusters from select organelles. The co-expression levels between genes from hyperLOPIT-defined subcompartment clusters belonging to the same organelle (excluding co-expression levels between genes from the same cluster) are shown as light-blue bars in histograms. The co-expression levels between the cluster members from the organelle and all the genes that are not members of the clusters from the organelle are shown as orange bars. For comparison, the co-expression levels between genes from the same cluster (as in **Figure S5*A***) are shown as thin lines.

The Y-axis shows the fraction of gene pairs. The X-axis shows Pearson correlation (the range is from –1 to 1) of non-normalized quantitative transcriptomics data retrieved from ToxoDB.org. Cohen's *d* values with *effect size* descriptors are shown above each plot.

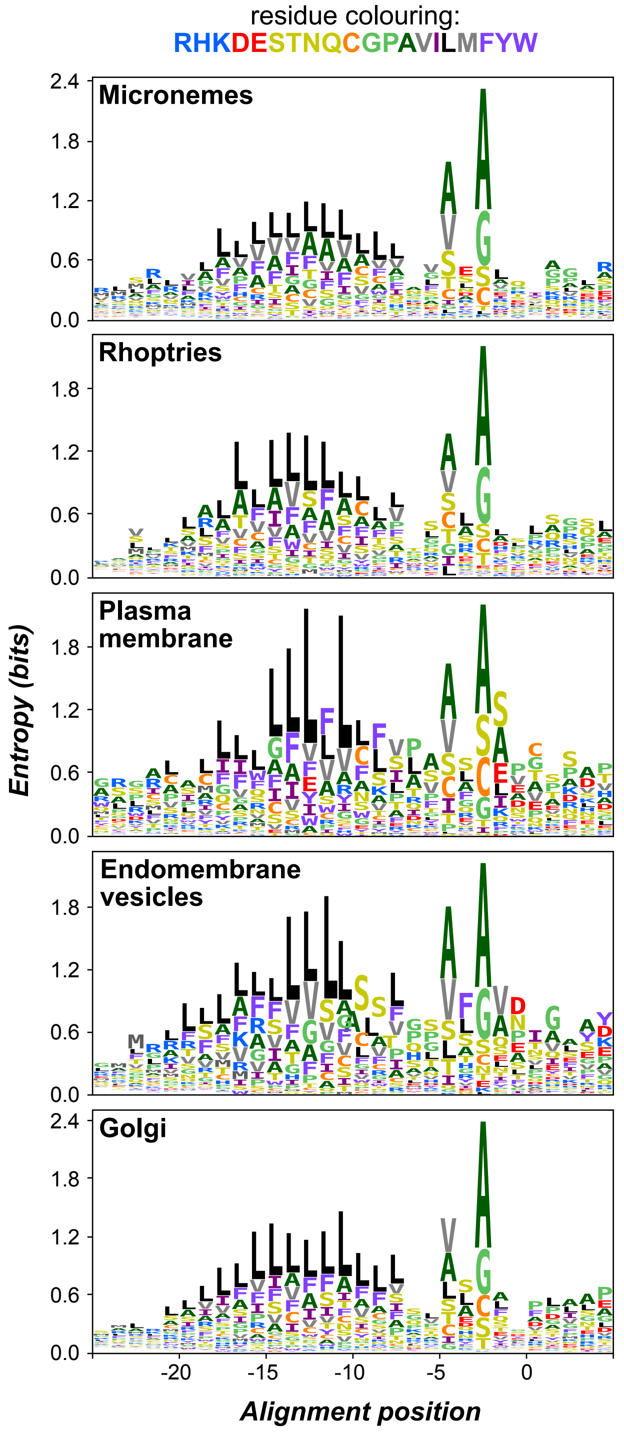

Figure S6. Logo plots of signal peptide (SP) sequences for select cohorts of *T. gondii* proteins, related to Figure 6.

The logo plots show positional abundances of amino acid residue types within and immediately downstream of the SP cleavage site. Proteins from hyperLOPIT-defined *T. gondii* compartment-specific sets and their close homologues from Apicomplexa were aligned anchored at the SP cleavage site (positon 0). Logo plots were generated after randomly sampling 1000 sequences for each data set from position-specific residue abundance probabilities calculated from dissimilarity-weighted sequences. The plots were generated in the same way as in **Figure 5*A***.

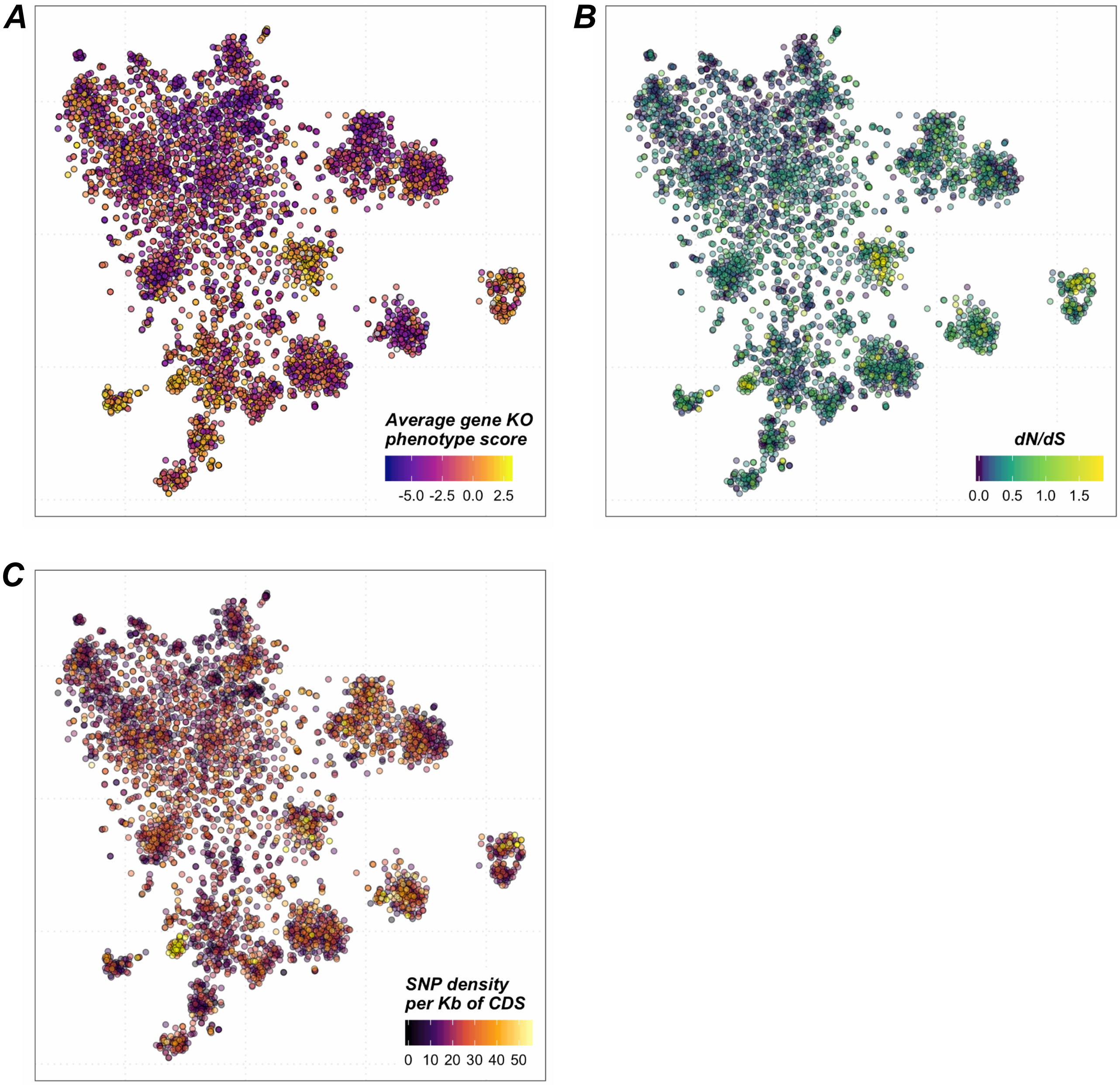

Figure S7. Mapping of the average CRISPR/Cas9-mediated gene knockout phenotype score (A), evolutionary selective pressure (B), and genetic polymorphism (C) on the 30plex hyperLOPIT t-SNE projection of *T. gondii* extracellular tachyzoite spatial proteome data, related to Figure 6.

A. Distribution of the average CRISPR/Cas9-mediated gene knockout phenotype score (Sidik et al., 2016). The range is from blue for essential genes to yellow for dispensable genes with the midpoint set at -2.4.

B. Distribution of the protein-average ratio of non-synonymous to synonymous point mutations (d_N/d_S) (Lorenzi et al., 2016). The scale is clipped at the 99-% quantile of the d_N/d_S range. Data points with extremely high (top-1%) d_N/d_S values are colored in yellow.

C. Distribution of the density of single nucleotide polymorphism (SNP) per Kb of protein-coding sequence (CDS) of genes. The SNP density data were retrieved from ToxoDB.org. As in ***A***, the scale is clipped at the 99-% quantile of the data range, with extremely high values (top-1%) shown in yellow.
